# App-based automated meal analysis in adults with type 1 diabetes using automated insulin delivery: a randomized controlled trial

**DOI:** 10.1016/j.eclinm.2025.103537

**Published:** 2025-10-08

**Authors:** Camillo D. Piazza, Céline I. Laesser, Lum Kastrati, Katharine Barnard-Kelly, Christos T. Nakas, David Herzig, Lia Bally

**Affiliations:** a Department of Diabetes, Endocrinology, Nutritional Medicine and Metabolism, Inselspital Bern, Bern University Hospital, University of Bern, Switzerland; b Southern Health NHS Foundation Trust, Southampton, UK; c School of Agricultural Sciences, Laboratory of Biometry, University of Thessaly, Volos, Greece; d Department of Clinical Chemistry, Inselspital, Bern University Hospital, University of Bern, Bern, Switzerland

**Keywords:** Type 1 diabetes, Nutritional management, Mobile health, Automated food analysis, Carbohydrate estimation, Insulin dosing support, Automated insulin delivery

## Abstract

**Background:**

Image-based automated meal analysis using smartphones has the potential to facilitate meal management in type 1 diabetes. We evaluated the glycaemic efficacy of SNAQ–an image-based automated meal analysis app–in adults using hybrid automated insulin delivery (AID) systems requiring carbohydrate entry for prandial insulin dosing.

**Methods:**

In this single-centre trial (NCT05671679) adults with type 1 diabetes on AID therapy were randomly assigned to using SNAQ—a commercial mobile app recognizing and quantifying food from images for meal management support—or continuing their usual meal management (control group) for 3 weeks. The primary endpoint was the change in the %time in range (TIR, 3.9–10.0 mmol/L). Following the first three weeks, SNAQ was also provided to the control group for evaluating the sustainability of benefits and usage across all participants.

**Findings:**

Twenty-two participants were randomized between March 14 and November 23 2023 to using SNAQ and 22 to control. At baseline, TIR was 75.4 ± 13.7% and 74.3 ± 12.7% in the intervention and control group, respectively. After three weeks, the baseline-adjusted difference in TIR between SNAQ (used 1.6 ± 0.8 per day) and control was 6.6 percentage points in favour of SNAQ (95% CI 2.9 to 10.3, P < 0.001). SNAQ further improved mean glucose (−0.54 mmol/L, CI −0.9 to −0.2, P = 0.004) and time above range (−6.3%, CI −10 to −2.7, P = 0.001). Time below range, total daily insulin dose, bolus frequency, nor carbohydrate entered into the pump did not significantly differ between groups. Post-discontinuation, the glycaemic benefits of SNAQ were not sustained. No study-related serious adverse events occurred.

**Interpretation:**

Short-term use of the automated meal analysis app SNAQ improved glucose control in adults with type 1 diabetes treated with AID.

**Funding:**

The study was supported by the EFSD/EUDF Digitalisation on Diabetes Care Research Grant and by the Diabetes Center Berne.

**Research in context**

**Evidence before this study**

Automated insulin delivery (AID) systems are now the standard of care in type 1 diabetes, helping people living with diabetes better manage their glucose while reducing burden. However, the majority of currently available systems are hybrid in nature and still require the person to enter carbohydrates manually into the system. Technological advancements in computer vision and artificial intelligence hold significant potential to address the persistent challenges of carbohydrate estimation. While numerous automated food recognition applications are available, few currently provide comprehensive quantification capabilities (e.g., carbohydrate content in grams). We searched PubMed for articles published before November 12, 2024 using the MeSH terms and/or key words for (“type 1 diabetes” OR “Diabetes Mellitus, Type 1” OR “T1D”) AND (“image processing, computer-assisted” OR “Artificial Intelligence” OR “computer assisted image analysis” OR “computer vision”) AND (“assessment, nutrition” OR “analysis, food” OR “meal analysis” OR “carbohydrate estimation” OR “food recognition”) AND (“Mobile Application” OR “Smartphone” OR “app”) AND (“Closed-Loop” OR “Artificial Pancreas” OR “Automated Insulin Delivery”). No eligible randomized controlled trials (RCTs) were identified. A previous crossover RCT of GoCARB (an image-based food analysis app) in sensor-augmented pump users showed reduced hyperglycemia and glucose variability versus standard care over one week.

**Added value of this study**

Our study, is to our knowledge, the first to compare the image-based automated food analysis SNAQ for meal management against usual care in AID users with type 1 diabetes. SNAQ is a commercial mobile app recognizing and quantifying food from images, providing carbohydrate estimates to support meal insulin dosing. Three-week SNAQ use improved glucose control, with no changes to insulin dosing or carbohydrate intake. SNAQ use decreased over time, and its glycaemic improvements did not persist after discontinuation.

**Implications of all the available evidence**

Automated meal analysis apps may improve postprandial glucose management in AID users with type 1 diabetes. Larger and longer studies are needed to elucidate mechanisms of action, optimize user engagement and evaluate efficacy across broader, more diverse patient populations.

## Introduction

Meal carbohydrate estimation for prandial insulin dose adjustment is a key component of an effective glucose management in type 1 diabetes,^1^^,^^2^ as carbohydrates are the main driver of postprandial glucose fluctuations.^3^^,^^4^ Thus, most current automated insulin delivery (AID) systems still require manual carbohydrate input for meal bolus delivery.

However, carbohydrate estimation is error-prone (20.9%–71.0% error rates^5^, ^6^, ^7^, ^8^) and burdensome for many individuals with type 1 diabetes.^9^

Automated meal analysis using images presents a promising approach to improving meal management in type 1 diabetes. While many mobile apps leverage artificial intelligence (AI)-driven image-based food recognition, few currently include portion size quantification.^10^, ^11^, ^12^, ^13^ These employ computer vision and depth sensing techniques to estimate food volume.^14^ SNAQ is a commercial app offering automated meal recognition and quantification from food images captured using depth-sensing smartphone cameras. In real-word testing with canteen meals, SNAQ estimated carbohydrates with a 44.3% mean absolute error, outperforming individuals with type 1 diabetes (71.0% error) confronted with identical meals.^7^ However, randomized trial evidence on SNAQ’s glycaemic impact and user acceptance in people with type 1 diabetes remains lacking.

Therefore, we conducted a randomized controlled trial (RCT) to evaluate SNAQ’s glycaemic impact and user acceptance in adults with type 1 diabetes using AID systems, comparing SNAQ to usual meal management.

## Methods

### Study design

This open-label RCT with a parallel group design was conducted at the University Hospital Bern, Switzerland, was approved by the Ethics Committee Bern (BASEC-ID 2022-02175), and pre-registered at clinicaltrial.gov (12/2022, NCT05671679, URL). Written informed consent was obtained from each participant before any study-related procedures.

The primary objective was to assess the efficacy of a three-week SNAQ app intervention in improving glycaemic control in adults with type 1 diabetes using AID. Further objectives included the evaluations of the sustainability of these effects and long-term app engagement.

**Fig. 1:**
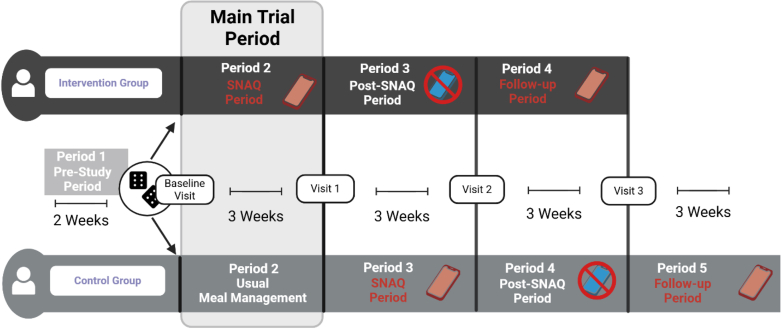
Study flow, participants were randomized at baseline to either receive SNAQ (intervention group) or continue with their usual meal management methods (control group) for 3 weeks. After the Main Trial Period, the intervention group entered the Post-SNAQ Period of 3 weeks, in which SNAQ was de-activated. The control group received access to SNAQ after the Main Trial Period and was asked to use it for 3 weeks. Thereafter, the control group entered the Post-SNAQ Period. Both groups underwent a final Follow-up Period with optional SNAQ use (Created with BioRender.com).

The analyses included four periods for the intervention group and five periods for the control group: Pre-Study Period (Period 1) for baseline glucose control assessment, a Main Trial Period (Period 2), in which participants were randomized to either use SNAQ for meal management (intervention) or continue with their usual meal management methods (control). As the control group did not use SNAQ during the Main Trial Period, these participants were provided with three-week access to the app thereafter (designated a Period 3 for the control group). Following the three weeks of SNAQ use, both groups entered the Post-SNAQ Period in which all participants discontinued SNAQ use to evaluate sustained effects (corresponding to Period 3 and 4 for intervention and control group, respectively). After the Post-SNAQ Period, all participants underwent a Follow-up Period with optional SNAQ use to assess long-term engagement (corresponding to Period 4 and 5 for intervention and control group, respectively). Providing SNAQ access to all participants following the randomized controlled period enabled evaluation of both sustained glycaemic effects and app acceptance across the entire study population, thereby increasing statistical power for these analyses. Fig. 1 illustrates the complete study flow, including all periods and specification when each group used or paused SNAQ use.

For the efficacy evaluation, glucose endpoints during the Main Trial Period were compared between the intervention (SNAQ) and control groups. For the sustainability evaluation of SNAQ use, data from both groups were combined and glucose endpoints in the 3 weeks following SNAQ discontinuation (Post-SNAQ Period) were compared against baseline (Period 1 and Period 2 for intervention and control group, respectively). Finally, optional app usage was tracked during the Follow-up Period.

### Study participants and randomization

Eligible participants were adults (≥18 years) with type 1 diabetes (duration ≥6 months) and HbA1c ≤ 12.0%, using commercially available AID systems in Switzerland for ≥three months. We excluded individuals with significant prior SNAQ exposure (>five days in the last three months), active pregnancy, or lactation (see Supplementary Table S1 for complete eligibility criteria).

After obtaining informed consent, we screened interested individuals for eligibility and collected most recent 14 days of baseline AID system data from eligible individuals. Randomization (1:1) to the use of SNAQ (intervention) or their usual meal management (control) employed minimization (MinimPy software^15^^,^^16^) with stratification by: baseline percentage time spent in glucose target range (3.9–10.0 mmol/L, <75 or >75%), HbA1c (<7.5 or ≥7.5%), and sex (male, female, these two options were available to participants).

### Procedures

Participants maintained their usual care AID systems and pre-study insulin (rapid or ultra-rapid acting) throughout all study phases. The study protocol imposed no dietary, travel, or activity restrictions during any phase. Participants managed their diabetes independently, without remote monitoring, therapeutic adjustments, or clinical supervision. A dedicated telephone helpline provided all participants with access to the study team for protocol-related support.

#### Intervention (The SNAQ application)

**Fig. 2:**
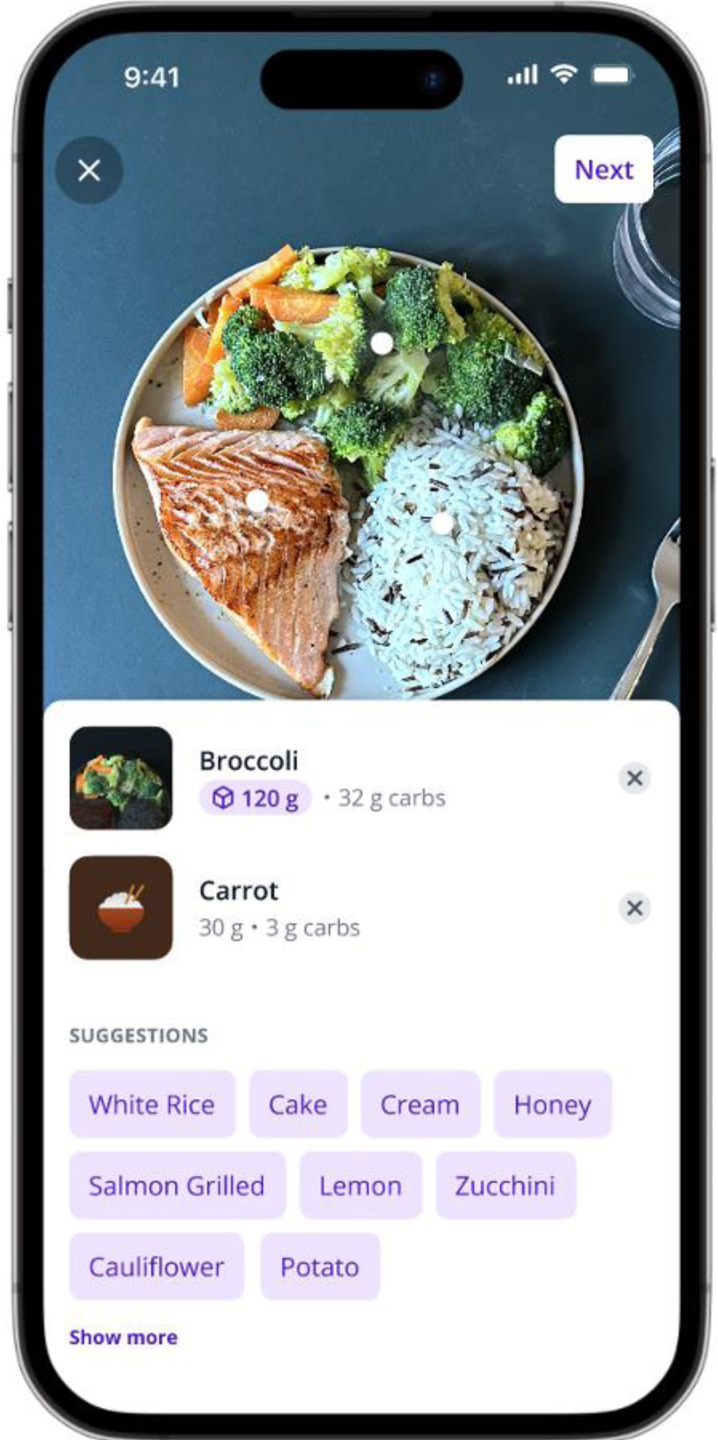
Automated meal macronutrient analysis combining image recognition with 3D reconstruction: This feature of the SNAQ application allows users to photograph their meals. Using AI, the application then provides food type suggestions for the individual components captured in the image.

The intervention consisted of using the automated meal analysis SNAQ smartphone application (Version 8 and 9, SNAQ AG, Winterthur, Switzerland, Fig. 2) with a LiDAR (3D) on LiDAR-equipped iPhone 12 Pro or newer iPhone models, enabling quantitative nutrient estimation instead of relying on standard serving sizes. With this set-up, SNAQ provided three meal management features: 1) Automated meal macronutrient analysis combining image recognition with 3D reconstruction (via LiDAR), 2) Barcode scanning for packaged foods available in Switzerland, with standard portions, and 3) Food look-up for standard portion nutritional information. The 3D analysis algorithm matched food volume measurements against a database containing food density and macronutrient information, to calculate energy, carbohydrates, fat, and protein content in kcal and grams (processing time: 20–30 s,^17^ see Supplementary Fig. S1).

All participants received standardized training on SNAQ functionalities and were recommended minimum usage of twice daily during the three weeks of SNAQ use. While all features were available, priority was given to automated (3D) nutrient estimation. Supplementary Table S2 details the step-by-step workflow for this analysis. For meal bolus calculations, participants could either accept the SNAQ’s carbohydrate estimates directly, or manually adjust the values based on their clinical judgement. Study-iPhones were provided to participants without compatible devices. The SNAQ application operated as a standalone system without integration with participants' AID systems. Specifically, no interface existed with any AID system’s bolus calculator, continuous glucose monitoring (CGM) data were not displayed, and insulin delivery histories were not visible within the app.

#### Main Trial Period

At enrolment, all participants completed a 16-item carbohydrate estimation quiz (meal pictures with a carbohydrate content ranging between 7 and 140 g; pictures are shown in the Supplementary Appendix) and a self-designed meal management questionnaire interrogating current strategies as well as perceived importance and burden. During the three-week Main Trial Period, the intervention group used the SNAQ app for meal management support while the control group maintained their standard meal management. Both groups repeated the carbohydrate estimation quiz at the end of the Main Trial Period.

#### Post-SNAQ period and sustainability assessment

After three weeks of SNAQ use, all participants completed a self-designed app satisfaction survey (corresponding to Period 2 and 3 in intervention and control group, respectively).

A three-weeks Post-SNAQ Period, with account deactivation, followed, to evaluate sustainability of effects following cessation of app usage.

#### Follow-up period with optional SNAQ use

After the Post-SNAQ Period, all participants regained access to SNAQ for optional use for 3 weeks. During this Follow-up Period, we tracked SNAQ engagement while glucose endpoints, as per the pre-registered protocol, were not assessed.

### Outcomes

#### Primary efficacy analysis

The primary endpoint was the change in the proportion of time when glucose was in range (TIR) between 3.9 and 10.0 mmol/L, as recorded by sensor glucose measurements.^18^ Secondary endpoints included changes in the overall time spent at glucose levels >10.0 mmol/L and <3.9 mmol/L and changes in time spent in postprandial period between 3.9 and 10.0 mmol/L, >10.0 mmol/L and <3.9 mmol/L. The postprandial period was defined as the 180-min following meal announcements (defined as carbohydrate entries ≥25 g in the AID system’s bolus calculator).

Other endpoints included changes in time spent between 3.9 and 7.8 mmol/L, >13.9 mmol/L and <3.0 mmol/L and changes in mean sensor glucose, glucose variability (measured by coefficient of variation, CV) and standard deviation of sensor glucose (SD). These metrics were analysed both overall and specifically during the postprandial period. Further, other endpoints included glucose management index (GMI), insulin metrics (total daily dose and manual bolus frequency), meal management behaviours (frequency and size of carbohydrate entries) and changes in carbohydrate estimation accuracy during the Main Trial Period.

#### Sustainability analysis

Sustainability effects of SNAQ use across all participants were evaluated using the same glucose and insulin endpoints as for the primary efficacy analysis, by comparing values during the pre-SNAQ versus post-SNAQ period.

#### Engagement with the SNAQ app and user experience

App engagement was quantified by analysing daily meal entries recorded in SNAQ during active and follow-up periods. Experience was further evaluated using the app satisfaction survey.

#### Safety

Safety was evaluated by monitoring the incidence of severe hypoglycaemia (defined as an event required third-party assistance), diabetic ketoacidosis, and any other study-related adverse or serious adverse events across all study periods.

### Statistical analysis

Sample size estimation was based on the primary endpoint. We assumed a mean improvement of 10% in time spent in the target glucose range when using SNAQ and a within-person standard deviation of 10%. To achieve a power of 80% at a two-sided alpha level of 0.05, 34 participants (17 per group) were required. To account for a dropout rate of 20%, we aimed to recruit a total of 44 participants. These assumptions were informed by preliminary data from subjects using the SNAQ application for their usual meal management.^19^

For the primary efficacy analysis, endpoints were compared between the two groups using linear models. All models were adjusted for the respective baseline values of the endpoints of interest. For the sustainability analysis, changes from the period prior to SNAQ use (Period 1 and 2 in intervention and control group, respectively) to Post-SNAQ Period (Period 3 and 4 in intervention and control group, respectively) were assessed using random effect models including group as random effect. Model assumptions were evaluated using graphical methods. For highly skewed data, extreme values were winsorized at the 5th and 95th percentiles to reduce the impact of outliers and approximate normality. For Time <3.0 mmol/L, which remained non-normal after winsorization, the Wilcoxon rank-sum test was used.

A linear mixed-effects model was used to assess differences across agreement categories between carbohydrate estimates provided by SNAQ and those entered into the AID system. The overall effect of agreement category was evaluated using a Wald chi-square test. Marginal means were compared pairwise using the Tukey method for P-value adjustment. In a subgroup analysis, we explored the potential role of sex-based differences. The test for interaction for sex based heterogeneous effects of the intervention was calculated using the Bland–Altman approach.^20^ Missing data were not imputed. Analyses were performed based on a modified intention-to-treat basis including all participants with at least 30% of sensor glucose data availability.

Categorical variables are presented as numbers and proportions, while continuous variables are reported mean ± SD, or as medians [25th and 75th percentile] for non-normal distributions. All instances of missing data are documented in parallel with their related outcome measures. Analyses were conducted with R version 4.3.0 (The R Foundation for Statistical Computing, Vienna, Austria).

### Role of the funding source

The funder of the study had no role in study design, data collection, data analysis, data interpretation, or writing of the report.

## Results

**Fig. 3:**
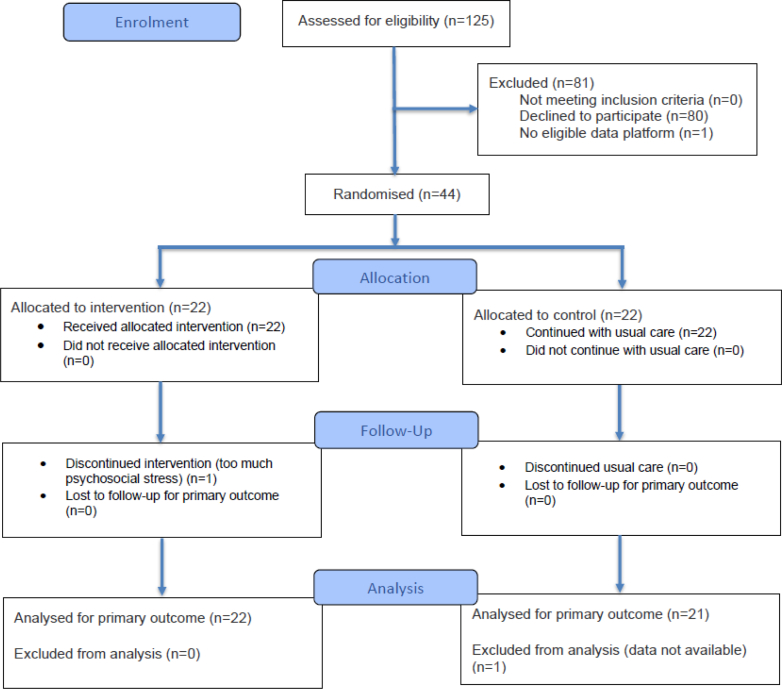
CONSORT flow of the primary endpoint (Main Trial Period). One participant in intervention group (left) discontinued intervention after 10 days. We analysed all participants with at least 30% of sensor glucose data, therefore we included all participants except one in control group for the primary endpoint (no AID system export data available). See Supplementary Table S3 for further details regarding analysis of other outcomes and study periods.

Between March 14 and November 23 2023, we enrolled and randomized 44 participants. One participant in the intervention group withdrew from the study after the second week of the Main Trial Period but was included in the analysis, having met the predefined threshold of ≥30% sensor glucose data. One participant in the control group had no AID system export data available. The primary endpoint analysis thus included data of 43 participants (see Fig. 3 for CONSORT 2025 Flow Diagram).

Two control group participants withdrew after completion of the Main Trial Period, and one additional intervention group participant dropped out during the Follow-up Period. In two participants, insulin metrics could not be calculated due to inaccessible insulin pump data. The sustainability analysis included data of 37 participants due to three withdrawals, two pump errors that deleted all pump memory, and two cases of missing data exports. Complete data availability for all analyses is detailed in Supplementary Table S3.

Table 1 presents baseline demographic and clinical characteristics for the overall study population, and by randomized group allocation. The study population (mean age 37 ± 15 years, range 18–69) comprised of 41% females and mean BMI of 25.5 ± 4.2 kg/m^2^. Participants used four different AID systems: Medtronic 780G (n = 21, 47%), mylife CamAPS FX (n = 13, 30%), Control-IQ (n = 9, 21%), and DBLG1 (n = 1, 2%). HbA1c at enrolment was 6.9% [6.4, 7.2] and time in range (3.9–10.0 mmol/L) was 74.9 ± 13.1%. Baseline carbohydrate estimation error (absolute and relative deviation from ground truth in a picture-based quiz) was 22.8 g [19.0, 26.5] and 44% [38.0, 54.0].

**Table 1:** Baseline participant characteristics overall and by treatment group.

	Total (n = 44)	Intervention (n = 22)	Control (n = 22)
Age [years]	36.8 ± 15	35.59 ± 14.7	38 ± 15.54
Female sex	19 (43%)	10 (46%)	9 (41%)
BMI [kg/m^2^]	25.5 ± 4.2	25.2 ± 4.6	25.9 ± 3.7
Baseline HbA1c [%]	6.9 [6.4, 7.2]	6.8 [6.4, 7.2]	6.9 [6.4, 7.4]
Percentage of time with glucose levels at 3.9–10.0 mmol/L [%]	74.9 ± 13.1	75.4 ± 13.7	74.3 ± 12.7
Duration of diabetes [years]	21.7 ± 14.1	19.1 ± 13	24.3 ± 15
Duration of AID therapy [years]	2 [1, 3]	2 [1, 3]	2 [1, 2]
**Type of AID system**			
DBLG1	1 (2%)	0 (0%)	1 (5%)
Medtronic 780G	21 (47%)	9 (40%)	12 (55%)
mylife CamAPS FX	13 (30%)	5 (23%)	8 (36%)
Control-IQ	9 (21%)	8 (36%)	1 (5%)
Daily insulin dose (units/kg/day)	0.6 [0.5, 0.8]	0.6 [0.5, 0.7]	0.7 [0.6, 0.9]
Absolute carbohydrate estimation error (g)	22.8 [19.0, 26.5]	23.6 [19.0, 26.2]	22.8 [19.5, 25.7]

Data are n(%), mean ± SD or median [25th and 75th percentile].

Carbohydrate estimation was perceived as a significant burden by 19/42 participants (45%) and 40/42 participants (95%) believed that new technologies could facilitate the process. Indicated methods for current meal management included conversion tables (5/42, 12%), experience (38/42, 91%), food labels (26/42, 62%), online resources or smartphone apps (different than the SNAQ app) (5/42, 12%), and weighing food (20/42, 48%). Avoiding certain meals because of difficulty determining carbohydrate content was reported by 7/42 participants (16%), most commonly for restaurant meals. 14/42 participants (33%) reported adjusting insulin doses based on non-carbohydrate macronutrients (e g fat/protein). Pizza, cakes, and pasta were identified as the most challenging meals to estimate carbohydrates.

Additional baseline characteristics and complete meal management questionnaire results are provided in Supplementary Tables S4 and S5, respectively.

**Fig. 4:**
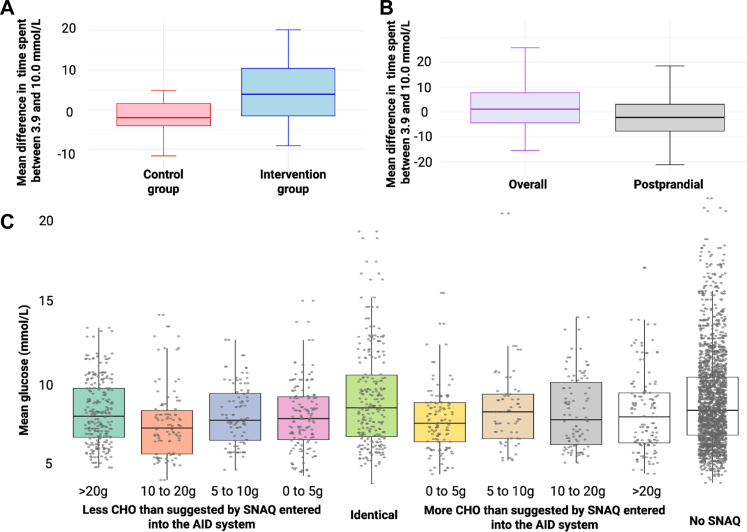
**A** Results form the Primary Efficacy Analysis: Mean difference in percentage time spent in range (TIR; 3.9–10.0 mmol/L) between intervention group (SNAQ use) and control group (usual meal management), adjusted for baseline TIR. **B** Results from the Sustainability Analysis: Mean difference in the percentage time spent in the glucose target range between 3.9 and 10.0 mmol/L between Post-SNAQ Period and the period preceding the SNAQ Period. Data shows differences of overall glucose control and postprandial (3 h following an announced meal) control. **C** Three hour post-meal mean glucose levels according to absolute deviations (in grams) between SNAQ-generated and participant-entered carbohydrate estimates. “No SNAQ” refers to meals where carbohydrates were entered into the bolus calculator without SNAQ assistance. Data from 38 participants during active SNAQ use period were included in this analysis (Created with BioRender.com).

Participants in the intervention group using SNAQ (mean engagement: 1.6 uses/day, corresponding to 42.9% of all announced meals) had a mean increase in TIR (3.9–10.0 mmol/L) of 3.9 ± 7.6 percentage points over the three-week Main Trial Period. In contrast, the control group experienced a mean decrease in TIR of 2.4 ± 5.3 percentage points. The resulting control-adjusted change (primary outcome), adjusted for baseline TIR, was a significant improvement of 6.6 percentage points in the intervention group compared with controls (95% CI 2.9 to 10.3, P < 0.001; Fig. 4, Panel A). Similarly, the intervention group significantly improved time spent at glucose levels 3.9–7.8 mmol/L, >10.0 mmol/L and >13.9 mmol/L and demonstrated significantly lower mean glucose, glucose variability, and GMI compared with the control group. No significant between-group differences were observed in time spent in either Level 1 (<3.9 mmol/L) or Level 2 (<3.0 mmol/L) hypoglycaemia. Analysis revealed no significant sex-by-treatment interaction effects on changes in TIR (P = 0.442) or other secondary outcomes. Results for primary, secondary and other glucose and insulin endpoints, as well as sex-by-treatment interaction effects, are detailed in Table 2 and Supplementary Table S6.

**Table 2:** Primary efficacy analysis: Effect of SNAQ use on glucose endpoints and insulin metrics.

	Intervention (n = 22)	Control (n = 21)	Control-adjusted change^a^	95% CI	P-value^b^
	Change	Change			
**Glucose levels over 24 h**					
Percentage of time with glucose levels at					
3.9–10.0 mmol/L [%]^c^	3.9 ± 7.6	−2.4 ± 5.3	6.6	2.9, 10.3	**<0.001**
3.9–7.8 mmol/L [%]	3.3 ± 8.7	−1.5 ± 5.1	4.6	0.3, 8.9	**0.036**
>10.0 mmol/L [%]	−4.2 [−9.9, 1.0]	1.4 [−1.2, 4.8]	−6.3	−10, −2.7	**0.001**
>13.9 mmol/L [%]	−0.2 [−4.2, 0.5]	0.8 [0.1, 2.3]	−3.6	−5.5, −1.6	**<0.001**
<3.9 mmol/L [%]	0.1 [−0.1, 0.4]	0.3 [−0.3, 0.5]	−0.2	−0.9, 0.4	0.446
<3.0 mmol/L [%]	0.0 [−0.1, 0.1]	0.0 [−0.1, 0.3]	−0.1	−0.2, 0.1	0.275
mean glucose [mmol/L]	−0.2 [−0.7, 0.2]	0.1 [−0.2, 0.4]	−0.5	−0.9, −0.2	**0.004**
SD of glucose [mmol/L]	−0.0 [−0.5, 0.1]	0.1 [−0.1, 0.3]	−0.3	−0.5, 0.0	**0.019**
CV of glucose [%]	−0.7 [−1.9, 1.5]	1.1 [−0.4, 2.4]	−1.1	−2.6, 0.3	0.129
GMI [%]	−0.2 ± 0.3	0.1 ± 0.2	−0.2	−0.4, −0.1	**0.004**
**Postprandial glucose levels**					
Percent of time with glucose levels					
3.9–10.0 mmol/L [%]	6.4 ± 13.8	−7.6 ± 13.3	13.4	6.2, 20.7	**<0.001**
3.9–7.8 mmol/L [%]	4.7 ± 12.0	−4.6 ± 14.0	8.5	1.1, 15.9	**0.025**
>10.0 mmol/L [%]	−2.9 [−15.8, 3.0]	8.2 [1.8, 12.9]	−13.9	−21.9, −5.9	**0.001**
>13.9 mmol/L [%]	−0.1 [−5.8, 0.4]	2.8 [−0.1, 10.3]	−10.4	−16.1, −4.8	**<0.001**
<3.9 mmol/L [%]	0.1 [−0.0, 1.9]	0.0 [−0.6, 1.2]	0.6	−0.7, 2	0.342
<3.0 mmol/L [%]	0.0 [0.0, 0.3]	0.0 [0.0, 0.2]	0.0	−0.2, 0.2	0.679^d^
Mean glucose [mmol/L]	−0.5 [−1.1, 0.3]	0.6 [−0.0, 1.2]	−1.3	−2.2, −0.5	**0.003**
SD of glucose [mmol/L]	−0.2 [−0.4, 0.0]	0.1 [−0.0, 0.4]	−0.3	−0.5, −0.1	**<0.001**
CV of glucose [%]	−0.8 [−2.2, 1.5]	0.8 [−1.4, 3.0]	−1.4	−3.7, 0.9	0.225
Peak glucose [mmol/L]	−0.6 [−1.9, 0.4]	0.8 [0.1, 1.4]	−1.8	−2.5, −1	**<0.001**
**Insulin metrics**					
Total insulin dose [IU/day]	−0.2 [−6.6, 1.3]	−0.0 [−7.4, 5.1]	−2.7	−7.8, 2.4	0.293
Manual insulin dose [IU/day]	0.6 [−1.0, 2.6]	0.1 [−1.8, 2.6]	−1.1	−3.9, 1.7	0.416
Automated insulin dose [IU/day]	−0.8 [−6.4, 0.6]	−0.1 [−5.9, 2.7]	−2.4	−5.9, 1.2	0.187
Manual bolus frequency [/day]	0.3 [−0.8, 1.3]	0.1 [−0.7, 1.0]	0.1	−0.4, 0.7	0.629
Carbohydrate input [g/day]	12.0 ± 23.8	−0.2 ± 23.3	12.9	−1.9, 27.7	0.085

Data are mean ± SD or median [25th and 75th percentile], GMI = glucose management index, CV = coefficient of variation. Change was calculated comparing the Pre-Study Period and Main Trial Period for each participant.

a Treatment effects are between-group differences in change from baseline (intervention minus control), estimated from linear regression models with the change score as the dependent variable and the baseline value of each outcome included as a covariate. P-values were obtained from the same linear models.

b P-values were obtained using two-tailed tests assessing non-equivalence.

c Primary endpoint.

d P-value was calculated using the Wilcoxon rank-sum test.

SNAQ's glycaemic benefits primarily resulted from postprandial glucose improvements (TIR +13.4 percentage points, 95% CI 6.2, 20.7, P < 0.001), with larger between-group differences observed in Period 2 compared to the overall analysis (Supplementary Table S7). Daily insulin use–including total, automated, and manual bolus doses as well as manual bolus frequency–showed no significant differences between groups. Similarly, there was no between-group difference in total daily carbohydrate entry. At the end of the three-week Main Trial Period neither group showed significant changes in carbohydrate estimation skills, and no between-group differences were observed (Supplementary Table S8).

The glycaemic benefits of SNAQ were not sustained after discontinuation (Fig. 4 Panel B). There was no significant change in TIR between the before and Post-SNAQ Periods (mean change: −0.5 percentage points; 95% CI, −3.4 to 2.5; P = 0.76). A small but statistically significant increase in time spent in Level 1 hypoglycaemia was observed (mean change: +0.4 percentage points; 95% CI, 0.1 to 0.6; P = 0.005). No significant changes were observed in other glycaemic metrics (all P > 0.49, Supplementary Table S9).

The 3D meal analysis feature was most frequently utilized (67% of uses), followed by lookup (27%) and barcode scanning (6%). App engagement showed a gradual decline over the study duration (−0.05 times/day for each additional day, 95% CI −0.06 to −0.04, P < 0.001, Supplementary Fig. S2).

After three weeks of SNAQ use, participant feedback revealed mixed perceptions of the app's effectiveness (Supplementary Table S10). 10/41 participants (24%) reported improved glucose control and 21/41 (51%) felt it enhanced their nutrition knowledge. Most 28/41 (68%) did not report any changes in dietary behaviours. Overall satisfaction ratings were evenly distributed across positive, neutral, and negative feedback. Although participants generally found the app user-friendly and visually appealing, some users (17/41, 42%) noted inaccuracies in its carbohydrate estimation, while smaller subgroups noted potential negative impact on social interactions (8/41, 20%) or heightened hypoglycaemia concerns (12/41, 29%). Notably, self-reported burden of carbohydrate estimation remained unchanged.

During the Follow-up Period when SNAQ was made available for optional use, only 5/39 participants (12.8%) utilized the app at least once over three weeks. Among these active users, median daily usage was 0.1 [0.1, 0.2] times per day.

The study recorded no instances of severe hypoglycaemia or diabetic ketoacidosis throughout all phases. A single serious adverse event occurred during Follow-up Period (pleural and pericardial effusion), which was determined to be unrelated to the study. Complete safety endpoint data across all study periods are presented in Supplementary Table S11.

Participants followed SNAQ’s suggestions in only 19.2% of cases, while entering lower carbohydrate amounts in 47.2% of instances.

Fig. 4 Panel C displays mean postprandial glucose levels stratified by deviation from SNAQ’s estimates. Time in range was lowest for meals without SNAQ use, with significantly lower values compared to several other agreement categories, including meals with >20 g, 20 g–10 g, 5 to 0 g, fewer and 0–5 g more than suggested (all P < 0.020). No other pairwise comparisons reached statistical significance. Complete glucose control metrics by carbohydrate estimate deviation are provided in Supplementary Table S12.

## Discussion

In this randomized controlled trial in adults with type 1 diabetes using AID systems, three weeks of SNAQ use—an automated meal analysis app—improved glucose control compared to standard meal management, demonstrating a 6.6 percentage-point increase in time in range (95% CI 2.9 to 10.3, P < 0.001). The intervention group showed improvements across multiple glycaemic endpoints, mainly attributable to improved postprandial glucose metrics, without significant differences in hypoglycemia or insulin dosing. While the treatment effect appears large for a short period of three weeks, it is important to note that the control group’s time in range decreased slightly compared to baseline potentially inflating the estimated effect of SNAQ (control-adjusted change). Nonetheless, the intervention group demonstrated a substantial improvement of 3.9 percentage points during SNAQ use.

The glycaemic benefits of SNAQ do not appear to be directly linked to its exact carbohydrate suggestions, as acceptance rates were low (19.2%) and a large proportion of user entries in bolus calculators deviated by > 20 g from the app’s estimate. Notably, neither the size nor direction of carbohydrate deviations significantly affected outcomes. While ground-truth carbohydrate of the assessed meals were unavailable, these findings suggest SNAQ’s benefits are independent of its estimation accuracy. Instead, we hypothesize that its value lies in prompting active meal engagement, promoting more conscious nutritional assessment and insulin bolusing. This observation is further supported by improved glucose levels following meals for which SNAQ was used, compared to meals without SNAQ. However, this difference may be partly confounded by factors such as meal types that are less compatible with SNAQ’s analysis features or by varying social contexts in which the app is less likely to be used. SNAQ’s comprehensive nutritional outputs (including energy, fat, and protein content)—whose glycaemic impacts are well-established^21^^,^^22^—may have further contributed to increased meal awareness.

Carbohydrate estimation skills showed no significant improvement following SNAQ use. Consistent with this finding, no sustained glycaemic benefits were observed after discontinuation of the app. While we note a statistically significant increase in time spent in Level 1 hypoglycaemia, the change is small, and the finding is unlikely to be attributable to the intervention and most likely reflects a chance fluctuation due to multiplicity. Despite a recommendation to use SNAQ at least twice daily, participants averaged 1.6 ± 0.8 uses per day over three weeks (corresponding to 38.6% of meals). Usage declined over time, with >75% of the participants discontinuing during optional follow-up. Despite positive user feedback on ease of use and reported willingness to use it future, our study found limited evidence that SNAQ reduces the burden of diabetes self-management, based on questionnaire responses. Usability challenges and time constraints are well-documented barriers to sustained engagement with diabetes management apps.^23^^,^^24^ In this study, SNAQ operated as a standalone application without integration into AID systems or connection to users' CGM data. Furthermore, the requirement for a separate LiDAR-equipped smartphone to access the 3D meal analysis feature in 34 participants likely created a usage barrier and potentially diminished perceived benefits. However, the development of adaptive insulin dosing algorithms (minimizing meal input to semi-quantitative announcements) and next-generation solutions aiming to eliminate meal input entirely^25^, ^26^, ^27^, ^28^, ^29^, ^30^ may reduce reliance on photo-based meal analysis, even with AID integration. Notably, while participants did not perceive subjective glucose improvements, the demonstrated objective benefits could potentially enhance adoption rates if users were made aware of these advantages.

Strengths of this study include its randomized controlled design and its focus on innovative technology for meal management—a critical daily challenge in diabetes self-care. Notably, it provides the first clinical evaluation of a commercially available image-based automated food analysis app for AID users. By enrolling users across multiple AID systems, the study’s findings demonstrate broad clinical applicability and reflect real-word diabetes management scenarios.

This study had several limitations. First, the relatively short intervention period and enrolment of predominantly well-controlled AID users may limit generalizability to populations with suboptimal glycaemic control or those using conventional pumps/multiple daily injections. Meal management tools like SNAQ could prove especially valuable for non-AID users, who cannot rely on automated insulin adjustments to mitigate carbohydrate counting inaccuracies. Second, randomization did not stratify by type of AID system, resulting in uneven distribution that could influence efficacy outcomes. Third, our findings specifically reflect SNAQ use with 3D-analysis-capable smartphones. Implementation of SNAQ with adjustable standard portions (as available on conventional smartphones) rather than automated 3D quantification could potentially yield different glycaemic outcomes. Fourth, the open-label design introduces potential performance bias, as active group participants' awareness of their intervention may have enhanced their motivation and glucose management efforts. Fifth, sustainability effects of SNAQ use were assessed across all participants in a single-arm design, without randomization, which limits inferring causal effects.

In conclusion, our study demonstrated that short-term use of SNAQ improved glucose control in people with type 1 diabetes treated with AID. The improvement likely stems not only from SNAQ’s automated carbohydrate suggestions—which were frequently adjusted—but also from the active engagement with meal bolus planning. Rather than replacing self-estimation entirely, SNAQ acted as a supportive tool, offering general trends rather than precise estimates. Sustained user engagement appears key to maximizing these benefits, highlighting the need for strategies to encourage long-term use. While our findings highlight the short-term benefits of SNAQ, further research is needed to assess its long-term impact on glucose control, advancing our understanding of automated meal analysis tools in type 1 diabetes management.

## Contributors

LB, DH co-designed the study and obtained funding. LB, DH and LK developed the protocol and were involved in the activities leading to regulatory approval. LB and CP recruited participants, provided patient care and interacted with study participants. LB, DH, CP, CIL, KBK and CTN carried out or supported data analysis, including the statistical analyses. LB, DH and CP wrote the manuscript. All authors critically reviewed the manuscript and contributed to the interpretation of the results. All authors critically reviewed the paper before publication, and had final responsibility for the decision to submit the research for publication. CP, DH and LB are the guarantor of this work and, as such, had full access to all the data in the study and take responsibility for the integrity of the data and the accuracy of the data analysis.

## Data sharing statement

The data generated and analysed in this study are not publicly available because of specifications in the consent forms. Data may be requested from the corresponding author under a data sharing agreement in accordance with local policies.

## Declaration of interests

LB reports having received speaker and advisory board honoraria from Eli Lilly, Dexcom, Novo Nordisk, Ypsomed and Roche Diabetes Care. All honoraria were allocated exclusively for research-related activities, in accordance with institutional policies. There are no personal financial interests or private benefits derived from these honoraria. LB serves as the president of Swiss Association for the Study of Obesity (ASEMO) and receives grants from the Swiss National Science Foundation, Swiss Innovation Agency, Medical Faculty of the University of Bern, Novo Nordisk Foundation and Bern MedTech Initiative. DH reports having received speaker honoraria from Ypsomed. CL received career funding from the Gottfried and Julia Bangerter-Rhyner Foundation and the Swiss Academy of Medical Sciences (SAMW). KBK reports having received research funding and lecture fees from Dexcom and Abbott. She further reports consultancy fees by Syneos Health and is founder and shareholder of Spotlight-AQ Ltd. The other authors declare no conflicts of interest. SNAQ AG supplied the app free of charge but was excluded from all research activities including design, execution, analysis, and scientific dissemination.

## Appendix A Supplementary data

Supplementary Figs. S1 and S2 and Tables S1–S12

Protocol_SUMMIT_V1_signed
