# Supplementary material for: App-based automated meal analysis in adults with type 1 diabetes using automated insulin delivery: a randomized controlled trial
Source: eClinicalMedicine. 2025 Oct 8;89:103537. doi: 10.1016/j.eclinm.2025.103537 (PMC12538901; doi:10.1016/j.eclinm.2025.103537)
Supplement: Supplementary Figs. S1 and S2 and Tables S1–S12 [file mmc1.docx]

**Supplementary Appendix**

**Supplementary Figures**

**Figure S1.** SNAQ’s automated food quantification workflow. Page 2

**Figure S2.** App engagement over the study duration Page 3

**Supplementary Tables**

**Table S1.** Eligibility criteria Page 4

**Table S2**. Step-by-step guide to obtaining automated nutrient estimations

with the SNAQ app Page 5

**Table S3.** Overview of data availability across all study periods Page 6

**Table S4.** Additional participant characteristics Page 7

Table S5. Results from the meal management questionnaire Page 8

**Table S6.** Sex-by-treatment interaction effects Page 12

**Table S7.** Postprandial glucose metrics over all study periods Page 13

**Table S8.** Glucose and insulin metrics and other endpoints over all study periods Page 14

**Table S9.** Sustainability Analysis: Change of glycaemic endpoints and
insulin metrics from Pre-SNAQ Period to the Post-SNAQ Period Page 15

Table S10. Results from the app satisfaction survey Page 16

Table S11. Safety endpoints Page 21

**Table S12.** Overview of agreement between SNAQ and entered CHO in
180min postprandial glucose values Page 22

**Other Supplementary Material**

Carbohydrate Estimation Quiz Page 23

**Figure S1. SNAQ’s automated food quantification workflow.**

**
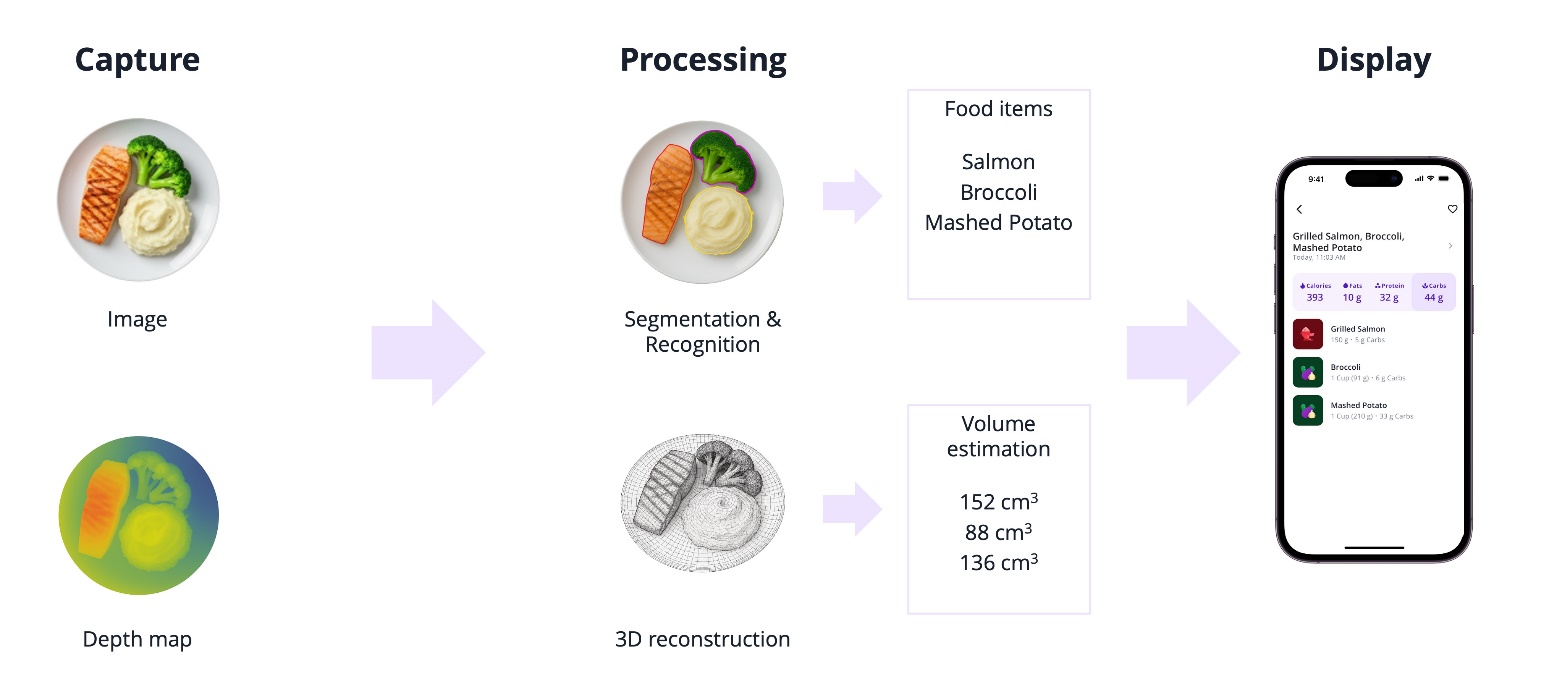
**

*Figure S1. SNAQ’s automated food quantification workflow employs a multi-stage computer vision pipeline integrating traditional image processing with modern deep learning techniques. This process initiates with scene capturing via the smartphone’s depth sensor. Subsequent computer vision and deep learning algorithms perform simultaneous food segmentation, recognition, and 3D reconstruction. Volume-to-weight conversion utilizes density parameters from SNAQ's food database, which also stores nutrient-per-gram values. This enables calculation and display of total nutrients (grams) and energy (kcal) per meal.*

**Figure S2. App engagement over the study duration**


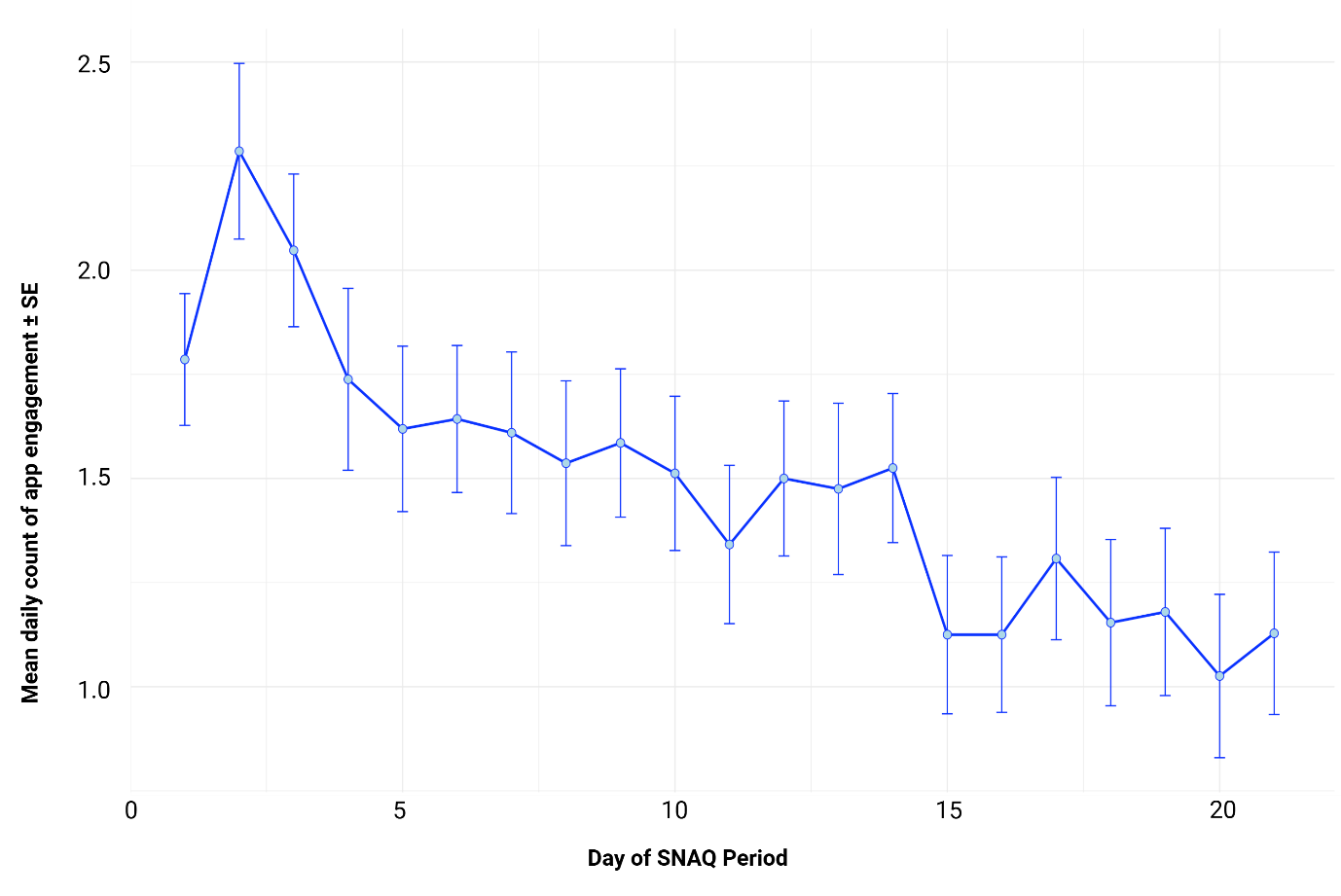


*Figure S2. App engagement showed a gradual decline over the study duration (-0·05 times/day for each additional day, 95% CI -0·06 to -0·04, P<0·001).*

**Table S1. Eligibility criteria**

|  | *Inclusion criteria* | *Exclusion criteria* |  |
| --- | --- | --- | --- |
|  | - Written informed consent | - Any physical or psychological disease or condition likely to interfere with the normal conduct of the study and interpretation of the study results |  |
|  | - Type 1 diabetes as defined by World Health Organisation for at least 6 months | - Previous use of SNAQ app for more than 5 days within the past 3 months |  |
|  | - Adults (aged 18 years or older) | - Self-reported pregnancy, planed pregnancy within next 3 months or breast-feeding |  |
|  | - Current use of a commercial automated insulin delivery system | - Severe visual impairment |  |
|  | - Glycated haemoglobin ≤12% (measured within the past 3 months) | - Severe hearing impairment |  |
|  | - Willingness to use the SNAQ app daily for ≥ 3 weeks | - Lack of reliable telephone facility for contact |  |
|  | - Willingness to follow study procedures and data sharing | - Concomitant participation in another trial that interferes with the normal conduct of the study and interpretation of the study results |  |
|  |  | - Limited German language proficiency |  |

*Table S1. Eligibility criteria*

**Table S2. Step-by-step guide to obtaining automated nutrient estimations with the SNAQ app.**

| **Step 1. Take a picture of your meal** | - Open the SNAQ app and tap the camera icon - Take a clear photo of your meal from directly above - Ensure good lighting and that all food items are visible - The app works best with well-lit, unobstructed views of the food |
| --- | --- |
| **Step 2. Review AI food recognition** | - SNAQ's AI automatically identifies food items in your photo - The app recognizes foods using computer vision and compares them against its extensive food database - Review the detected items and make corrections if needed - You can add missing ingredients or modify identified foods |
| **Step 3. Confirm portion sizes** | - The app uses 3D modelling to calculate volume and estimate weight - Verify the suggested portion sizes and adjust if necessary |
| **Step 4. Get instant nutritional breakdown** | - The app provides immediate calculations for:   - Carbohydrates (grams)   - Protein (grams)   - Fat (grams)   - Energy (kcal) - The nutritional analysis typically appears within seconds |

*Table S2. Step-by-step guide to obtaining automated nutrient estimations with the SNAQ app. The process comprises four steps to generate automated nutrient quantification from food photos. For alternative methods (manual search and barcode scanning), nutrient estimates derive from standardized portion sizes, which users may adjust manually. All three approaches—image analysis, manual search, and barcode scanning—can be combined within a single meal entry. Participants were explicitly informed that automated nutrient estimations carry inherent accuracy limitations and should be verified against personal judgements.*

**Table S3. Overview of data availability across all study periods**

|  |  | **Pre-Study Period** | | **Main Trial Period** | | **Post-SNAQ Period** | **SNAQ Period** | **Post-SNAQ Period** | **Follow-up Period** | |  |
| --- | --- | --- | --- | --- | --- | --- | --- | --- | --- | --- | --- |
|  |  | Intervention (n = 22) | Control (n = 22) | Intervention (n = 22) | Control (n = 22) | Intervention (n = 21) | Control (n = 20) | Control (n = 20) | Intervention (n = 20) | Control (n = 20) |  |
|  |  | SNAQ | Usual care | SNAQ | Usual care | SNAQ off | SNAQ | Usual care | Usual care | Usual care |  |
|  | **Participant information** | 22 | 22 | - | - | - | - | - | - | - |  |
|  | **CGM overall data** | 22 | 21^‡^ | 22 | 21^‡^ | 21^#^ | 18^††^ | 16^¶¶^ | - | - |  |
|  | **CGM postprandial data** | 19^*^ | 20^§^ | 20^¶^ | 20^§^ | 19^**^ | 18^††^ | 16^¶¶^ |  |  |  |
|  | **Manual insulin data** | 22 | 21^‡^ | 22 | 21^‡^ | 21^#^ | 18^††^ | 16^¶¶^ | - | - |  |
|  | **Automated insulin data** | 22 | 19^\|\|^ | 22 | 19^\|\|^ | 21^#^ | 16^‡‡^ | 14^##^ | - | - |  |
|  | **Total daily insulin data** | 22 | 19^\|\|^ | 22 | 19^\|\|^ | 21^#^ | 16^‡‡^ | 14^##^ | - | - |  |
|  | **SNAQ data** | - | - | 22 | - | - | 19^§§^ |  | 20^***^ | 19^§§^ |  |
|  | **Meal management questionnaire** | 21^†^ | 21^†^ | - | - | - | - | - | - | - |  |
|  | **CHO estimation quiz** | 19^†^ | 20^†^ | 16^†^ | 19^†^ | - | - | - | - | - |  |
|  | **App satisfaction survey** | - | - | 21^†^ | - | - | 20^\|\|\|\|^ | - | - | - |  |

*Table S3. Overview of data availability, numbers in parentheses refer to active participants (n) in corresponding study period, CHO = carbohydrate * 3 participants without eligible user entries in bolus calculators* † *missing data without dropouts* ‡ *1 dropout before AID system data collection* § *1 dropout before AID system data collection and 1 participant without eligible user entries in bolus calculators* || *1 dropout before AID system data collection, 2 participants without an eligible csv file for the automated insulin data* ¶ *2 participants without eligible user entries in bolus calculators* # *1 dropout* ** *1 dropout and 2 participants without eligible user entries in bolus calculators* †† *2 dropouts and 2 pump errors* ‡‡ *2 dropouts, 2 pump errors and 2 participants without an eligible csv file for automated insulin data* §§ *2 dropouts and 1 user with SNAQ account issues* |||| *2 dropouts* ¶¶ *2 dropouts, 2 pump errors and 2 participants without available AID system data* ## *2 dropouts, 2 pump errors, 2 participants without available AID system data and 2 participants without an eligible csv file for the automated insulin data* *** *1 dropout and 1 participant that did not want to participate in the optional Follow-up Period*

**Table S4. Additional participant characteristics**

|  |  | *Total (n = 44)* | *Intervention (n = 22)* | *Control (n = 22)* |  |
| --- | --- | --- | --- | --- | --- |
|  | **Race or ethnic group – no** |  |  |  |  |
|  | White European | 43 (97%) | 21 (95%) | 22 (100%) |  |
|  | Other | 1 (2%) | 1 (5%) | 0 (0%) |  |
|  | **Employment status** |  |  |  |  |
|  | Student | 5 (11%) | 2 (9%) | 3 (14%) |  |
|  | Unemployed | 8 (18%) | 5 (23%) | 3 (14%) |  |
|  | Employed | 29 (66%) | 14 (64%) | 15 (68%) |  |
|  | Retired | 2 (5%) | 1 (5%) | 1 (5%) |  |
|  | **Education** |  |  |  |  |
|  | Secondary school | 5 (11%) | 4 (18%) | 1 (5%) |  |
|  | Apprenticeship | 17 (39%) | 6 (27%) | 11 (50%) |  |
|  | High school | 9 (21%) | 3 (14%) | 6 (27%) |  |
|  | University degree | 13 (30%) | 9 (41%) | 4 (18%) |  |
|  | **Participants with cardio-renal-metabolic comorbidities** | 36 (82%) | 16 (73%) | 20 (91%) |  |
|  | **Cardio-renal-metabolic cormobidities** |  |  |  |  |
|  | Dyslipidemia | 20 (46%) | 7 (32%) | 13 (59%) |  |
|  | Hypertension | 10 (23%) | 5 (23%) | 5 (23%) |  |
|  | Obstructive sleep apnea | 1 (2%) | 0 (0%) | 1 (5%) |  |
|  | Coronary Heart Disease | 2 (5%) | 1 (5%) | 1 (5%) |  |
|  | Peripheral Artery Disease | 4 (9%) | 2 (9%) | 2 (9%) |  |
|  | Nephropathy | 5 (11%) | 2 (9%) | 3 (14%) |  |
|  | Neuropathy | 8 (18%) | 3 (14%) | 5 (23%) |  |
|  | Retinopathy | 10 (23%) | 4 (18%) | 6 (27%) |  |
|  | **Smoking status: current** | 6 (14%) | 2 (9%) | 4 (18%) |  |
|  |  |  |  |  |  |
|  | **Rapid/ultra-rapid insulin** |  |  |  |  |
|  | Aspart | 7 (16%) | 3 (14%) | 4 (18%) |  |
|  | Faster aspart | 22 (50%) | 10 (46%) | 12 (55%) |  |
|  | Faster lispro | 14 (32%) | 9 (41%) | 5 (23%) |  |
|  | Glulisine | 1 (2%) | 0 (0%) | 1 (5%) |  |
|  | **Type of continuous glucose monitoring (CGM)** |  |  |  |  |
|  | Dexcom G6 | 23 52%) | 13 (59%) | 10 (46%) |  |
|  | Guardian 4 | 21 (48%) | 9 (41%) | 12 (55%) |  |
|  |  |  |  |  |  |
|  | **Other glucose-lowering drugs** |  |  |  |  |
|  | Metformin | 2 (5%) | 0 (0%) | 2 (9%) |  |
|  | SGLT2 inhibitors | 1 (2%) | 1 (5%) | 0 (0%) |  |
|  | GLP-1 receptor agonists | 2 (5%) | 0 (0%) | 2 (9%) |  |

*Table S4. Values are n (%). Additional participant characteristics, numbers in parentheses refer to the number of participants (n) contributing to the analysis. See Table S3 for further details.*

**Table S5. Results from the meal management questionnaire**

|  | Do you feel that estimating carbohydrates puts an additional burden on your diabetes management? | Absolute (n = 42) | Percentage [%] |  |
| --- | --- | --- | --- | --- |
|  | No | 23 | 54·8 |  |
|  | Yes | 19 | 45·2 |  |
|  |  |  |  |  |
|  | Do you find it difficult to accurately estimate the carbohydrate content? | Absolute (n = 42) | Percentage [%] |  |
|  | No | 4 | 9·5 |  |
|  | Yes | 9 | 21·4 |  |
|  | Sometimes | 29 | 69 |  |
|  |  |  |  |  |
|  | How would you rate your own carbohydrate estimation ability? | Absolute (n = 42) | Percentage [%] |  |
|  | Less than 5% | 0 | 0 |  |
|  | 5% to 10% | 4 | 9·5 |  |
|  | 10% to 20% | 21 | 50 |  |
|  | 20% to 30% | 12 | 28·6 |  |
|  | More than 30% | 5 | 11·9 |  |
|  |  |  |  |  |
|  | Do you think that new technologies could facilitate the estimation of carbohydrates? | Absolute (n = 42) | Percentage [%] |  |
|  | No | 2 | 4·8 |  |
|  | Yes | 40 | 95·2 |  |
|  |  |  |  |  |
|  | How do you normally estimate the carbohydrate content of a meal? (multiple answers possible) | Absolute (n = 42) |  |  |
|  | Conversion tables | 5 |  |  |
|  | Experience/Estimation | 38 |  |  |
|  | Food labelling | 26 |  |  |
|  | Other: Counting Pieces | 1 |  |  |
|  | Web or smartphone app | 5^*^ |  |  |
|  | Weighing | 20 |  |  |
|  |  |  |  |  |
|  | Have you attended to a Carbohydrate Estimation Course? | Absolute (n = 42) | Percentage [%] |  |
|  | No | 14 | 33·3 |  |
|  | Yes | 28 | 66·7 |  |
|  |  |  |  |  |
|  | When did you attend to a Carbohydrate Estimation Course? (multiple answers possible) | Absolute (n = 42) | Percentage [%] |  |
|  | Less than 1 year ago | 1 | 3·4 |  |
|  | Less than 2 years ago | 1 | 3·4 |  |
|  | Less than 3 months ago | 2 | 6·9 |  |
|  | Less than 5 years ago | 3 | 10·3 |  |
|  | More than 1 year ago | 9 | 31 |  |
|  | More than 2 years ago | 2 | 6·9 |  |
|  | More than 5 years ago | 11 | 37·9 |  |
|  |  |  |  |  |
|  | Do you find that the hassle of estimating carbohydrates stops you from eating certain foods? | Absolute (n = 42) | Percentage [%] |  |
|  | No | 35 | 83·3 |  |
|  | Yes | 7 | 16·7 |  |
|  |  |  |  |  |
|  | Where? | Absolute (n = 6) | Percentage [%] |  |
|  | Self-made meals | 0 | 0 |  |
|  | Meals in a restaurant | 4^†^ | 66·7 |  |
|  | Other | 2^‡^ | 33·3 |  |
|  |  |  |  |  |
|  | Do you feel that food components other than carbohydrate affect your glucose levels after eating? | Absolute (n = 42) | Percentage [%] |  |
|  | No | 9 | 21·4 |  |
|  | Yes | 33 | 78·6 |  |
|  |  |  |  |  |
|  | Which components? (multiple answers possible) | Absolute (n = 42) |  |  |
|  | Fat | 27 |  |  |
|  | Fiber | 13 |  |  |
|  | Protein | 12 |  |  |
|  | Quality of the carbohydrates | 26 |  |  |
|  |  |  |  |  |
|  | Do you currently take into account other food components (e g fat, protein or fibre) in addition to the carbohydrate content when calculating your insulin dose? | Absolute (n = 42) | Percentage [%] |  |
|  | No | 28 | 66·7 |  |
|  | Yes | 14 | 33·3 |  |
|  |  |  |  |  |
|  | Points taken into account when applying insulin (open, non-mandatory question) |  |  |  |
|  | “Late insulin application” |  |  |  |
|  | “Round up or down, depending on protein and fat content” |  |  |  |
|  | “Less insulin at the start of the meal with the option to add more insulin later” |  |  |  |
|  | “Estimate food according to carb dynamic and experience about insulin requirements, not only according to carb content” |  |  |  |
|  | “I use the delay option of the Ypsopump for high fat, protein or fibre intake” |  |  |  |
|  | “I take fatty meals into account as these are absorbed a little more slowly” |  |  |  |
|  | “On the basis of a nutrition plan that I was given during nutrition therapy and examinations regarding my metabolism (number of meals per day and their contents)” |  |  |  |
|  | “For very greasy dishes such as raclette or fondue” |  |  |  |
|  | “Fat fondue difficult to estimate correctly)” |  |  |  |
|  | “Fat, simple/complex carbohydrates” |  |  |  |
|  |  |  |  |  |
|  | Name the 3 food items that are most challenging to estimate for you |  |  |  |
|  | Pizza | 14 |  |  |
|  | Cakes | 9 |  |  |
|  | Pasta | 7 |  |  |
|  | Casserole | 6 |  |  |
|  | Desserts/Sweets | 6 |  |  |
|  | Restaurant | 6 |  |  |
|  | Rice | 5 |  |  |
|  | Different Components | 4 |  |  |
|  | Fondue | 4 |  |  |
|  | Fruits | 4 |  |  |
|  | Lasagne | 4 |  |  |
|  | Sauces | 4 |  |  |
|  | Brunch | 3 |  |  |
|  | French Fries | 3 |  |  |
|  | Soups | 3 |  |  |
|  | Stew | 3 |  |  |
|  | Müesli | 2 |  |  |
|  | Bread | 2 |  |  |
|  |  |  |  |  |
|  | How important is nutrition for your general health? | Absolute (n = 42) | Percentage [%] |  |
|  | Not important | 3 | 7·1 |  |
|  | Something important | 9 | 21·4 |  |
|  | Important | 17 | 40·5 |  |
|  | Very important | 13 | 31 |  |
|  |  |  |  |  |
|  | Do you pay attention to your diet for reasons other than blood sugar control? | Absolute (n = 42) | Percentage [%] |  |
|  | No | 15 | 35·7 |  |
|  | Yes | 27 | 64·3 |  |
|  |  |  |  |  |
|  | What reasons? (multiple answers possible) | Absolute (n = 42) | Percentage [%] |  |
|  | Athletic performance | 5 | 10·4 |  |
|  | Other | 5 | 10·4 |  |
|  | Prevention of cardiovascular diseases | 7 | 14·6 |  |
|  | Weight Control | 13 | 27·1 |  |
|  | Well-being | 18 | 37·5 |  |
|  |  |  |  |  |
|  | What other reasons to pay attention to your diet? | Absolute (n = 5) |  |  |
|  | «Arthrosis» | 1 |  |  |
|  | «Coeliac Disease» | 2 |  |  |
|  | «Concentration/Cognitive Aspects» | 2 |  |  |
|  |  |  |  |  |
|  | To what extent did you feel fatigued by estimating carbohydrates? | Absolute (n = 42) | Percentage [%] |  |
|  | Not at all | 11 | 26·2 |  |
|  | Little | 15 | 35·7 |  |
|  | A bit | 13 | 31 |  |
|  | A lot | 3 | 7·1 |  |
|  | Extremely | 0 | 0 |  |
|  |  |  |  |  |
|  | How concerned were you while using the app that an inaccurate estimation of carbohydrates could lead to a dangerous event? | Absolute (n = 42) | Percentage [%] |  |
|  | Not at all | 10 | 23·8 |  |
|  | Little | 18 | 42·9 |  |
|  | A bit | 7 | 16·7 |  |
|  | A lot | 6 | 14·3 |  |
|  | Extremely | 1 | 2·4 |  |
|  |  |  |  |  |
|  | How concerned were you while using the app that inaccurate estimation of carbohydrates could lead to long-term complications? | Absolute (n = 42) | Percentage [%] |  |
|  | Not at all | 7 | 16·7 |  |
|  | Little | 8 | 19 |  |
|  | A bit | 11 | 26·2 |  |
|  | A lot | 14 | 33·3 |  |
|  | Extremely | 2 | 4·8 |  |
|  |  |  |  |  |
|  | How worried are you that counting carbs gets in your way, limits your spontaneity or disturbs other people you're with? | Absolute (n = 42) | Percentage [%] |  |
|  | Not at all | 18 | 42·9 |  |
|  | Little | 10 | 23·8 |  |
|  | A bit | 7 | 16·7 |  |
|  | A lot | 5 | 11·9 |  |
|  | Extremely | 2 | 4·8 |  |
|  |  |  |  |  |
|  | To what extent did you feel restricted in your social life by the limited choice of food and the difficulty of appreciating carbohydrates? | Absolute (n = 42) | Percentage [%] |  |
|  | Not at all | 20 | 47·6 |  |
|  | Little | 12 | 28·6 |  |
|  | A bit | 6 | 14·3 |  |
|  | A lot | 4 | 9·5 |  |
|  | Extremely | 0 | 0 |  |
|  |  |  |  |  |

*Table S5. Results from the meal management questionnaire, numbers in parentheses refer to the number of participants (n) contributing to the analysis. See Table S3 for further details. * Google Research, Lifesum, Fddb, YAZIO, DIABTREND † fat content is a problem in restaurants, stew, cakes, pizza, spaghetti/tortellini, complex dishes (hidden carbs): sauce* ‡ *pizza, pasta, stew, sweet dishes, Coca Cola, donut*

**Table S6. Sex-by-treatment interaction effects**

|  |  | **Mean Difference (95%-CI)** | | **Difference** | **95%-****CI** | **P-value** | |  |
| --- | --- | --- | --- | --- | --- | --- | --- | --- |
|  |  | Males (n = 25) | Females (n = 18) |  |  |  |  | |
|  | Percent of time with sensor glucose level |  |  |  |  |  | |  |
|  | 3·9 - 10·0mmol/l [%] | -5·5 (-10·6, -0·5) | -8·7 (-15, -2·5) | -3·2 | -11·2, 4·9 | 0·442 | |  |
|  | 3·9 - 7·8 mmol/l [%] | -3·0 (-9·2, 3·2) | -7·0 (-13·8, -0·2) | -4·0 | -13·2, 5·2 | 0·397 | |  |
|  | >10·0 mmol/l [%] | 5·2 (-0·1, 10·5) | 8·3 (2·4, 14·3) | 3·1 | -4·9, 11·1 | 0·449 | |  |
|  | >13·9 mmol/l [%] | 2·7 (0·6, 4·9) | 5·0 (1·6, 8·4) | 2·3 | -1·8, 6·3 | 0·273 | |  |
|  | <3·9 mmol/l [%] | 0·0 (-0·8, 0·9) | 0·4 (-0·4, 1·2) | 0·4 | -0·8, 1·6 | 0·528 | |  |
|  | <3·0 mmol/l [%] | 0·0 (-0·1, 0·2) | 0·3 (-0·1, 0·7) | 0·3 | -0·1, 0·7 | 0·167 | |  |
|  | Mean glucose [mmol/L] | 0·5 (-0·1, 1·0) | 0·7 (0·2, 1·2) | 0·2 | -0·5, 0·9 | 0·587 | |  |
|  | SD of glucose [mmol/L] | 0·5 (0·0, 0·9) | 0·3 (-0·2, 0·9) | -0·1 | -0·8, 0·6 | 0·702 | |  |
|  | CV of glucose [%] | 3·6 (-0·1, 7·2) | 1·1 (-3·4, 5·6) | -2·4 | -8·2, 3·4 | 0·412 | |  |

*Table S6. Sex-by-treatment interaction effects. Sex-specific differences of the intervention effect was calculated using the Bland–Altman approach, numbers in parentheses refer to the number of participants (n) contributing to the analysis. See Table S3 for further details.*

**Table S7. Postprandial glucose metrics over all study periods**

|  |  | **Pre-Study Period** | | **Main Trial Period** | | **Post-SNAQ Period** | **SNAQ Period** | **Post-SNAQ Period** | |  |
| --- | --- | --- | --- | --- | --- | --- | --- | --- | --- | --- |
|  |  | Intervention (n = 19) | Control (n = 20) | Intervention (n = 20) | Control (n = 20) | Intervention (n = 19) | Control (n = 18) | Control (n = 16) |  | |
|  |  | Usual care | Usual care | SNAQ | Usual care | SNAQ off | SNAQ | SNAQ off |  | |
|  | Percent of time with sensor glucose level |  |  |  |  |  |  |  | |  |
|  | 3·9 - 10·0mmol/l [%] | 64·5 ± 21·5 | 65·9 ± 17·3 | 68·8 ± 15·0 | 58·3 ± 19·0 | 66·1 ± 15·7 | 62·0 ± 15·1 | 51·1 ± 16·6 | |  |
|  | 3·9 - 7·8 mmol/l [%] | 38·2 ± 18·8 | 40·4 ± 16·5 | 42·0 ± 12·9 | 35·8 ± 17·8 | 40·9 ± 16·3 | 37·8 ± 16·2 | 28·0 ± 16·3 | |  |
|  | >10·0 mmol/l [%] | 28·2 [16·8, 46·3] | 31·0 [20·0, 49·1] | 28·1 [16·6, 37·7] | 43·2 [21·2, 53·5] | 32·2 [18·9, 42·5] | 35·7 [25·8, 48·3] | 51·4 [39·4, 60·1] | |  |
|  | >13·9 mmol/l [%] | 3·8 [1·4, 11·4] | 4·3 [1·6, 16·4] | 3·9 [0·9, 7·4] | 12·8 [3·2, 21·1] | 5·3 [1·7, 12·6] | 7·9 [3·7, 12·4] | 18·5 [8·6, 28·7] | |  |
|  | <3·9 mmol/l [%] | 0·6 [0·2, 2·5] | 1·7 [0·3, 2·8] | 0·7 [0·2, 3·6] | 1·7 [0·3, 3·5] | 1·2 [0·1, 2·3] | 2·0 [0·6, 2·7] | 1·9 [0·3, 4·2] | |  |
|  | <3·0 mmol/l [%] | 0·0 [0·0, 0·2] | 0·1 [0·0, 0·3] | 0·1 [0·0, 0·3] | 0·2 [0·0, 0·7] | 0·0 [0·0, 0·2] | 0·2 [0·0, 0·5] | 0·1 [0·0, 0·7] | |  |
|  | Mean glucose [mmol/L] | 9·2 ± 1·8 | 9·0 ± 1·6 | 8·7 ± 1·4 | 9·7 ± 2·0 | 9·0 ± 1·3 | 9·3 ± 1·4 | 10·2 ± 1·5 | |  |
|  | SD of glucose [mmol/L] | 1·6 [1·4, 1·8] | 1·7 [1·3, 1·9] | 1·4 [1·3, 1·7] | 1·7 [1·6, 2·1] | 1·5 [1·4, 1·8] | 1·7 [1·5, 1·9] | 2·0 [1·7, 2·6] | |  |
|  | CV of glucose [%] | 17·4 [16·4, 20·6] | 18·5 [16·7, 22·4] | 17·5 [15·3, 20·6] | 19·7 [17·7, 21·6] | 17·7 [16·7, 19·9] | 19·1 [17·3, 20·8] | 20·7 [18·5, 24·3] | |  |
|  | Peak glucose [mmol/l] | 11·2 [10·4, 13·4] | 11·4 [10·6, 13·4] | 11·1 [10·1, 11·7] | 12·8 [11·0, 14·0] | 11·5 [10·7, 12·8] | 12·3 [11·6, 13·6] | 14·3 [12·0, 15·5] | |  |

*Table S7. Data are mean ± SD or median [25th and 75th percentile], CV = coefficient of variation. Numbers in parentheses refer to the number of participants (n) contributing to the analysis. See Table S3 for further details.*

**Table S8. Glucose and insulin metrics and other endpoints over all study periods**

|  |  | **Pre-Study Period** | | **Main Trial Period** | | **Post-SNAQ Period** | **SNAQ Period** | **Post-SNAQ Period** | |  |
| --- | --- | --- | --- | --- | --- | --- | --- | --- | --- | --- |
|  |  | Intervention (n = 22) | Control (n = 21) | Intervention (n = 22) | Control (n = 21) | Intervention (n = 21) | Control (n = 18) | Control (n = 16) |  | |
|  |  | Usual care | Usual care | SNAQ | Usual care | SNAQ off | SNAQ | SNAQ off |  | |
|  | Percent of time with sensor glucose level at |  |  |  |  |  |  |  | |  |
|  | 3·9 - 10·0mmol/l [%] | 75·4 ± 13·7 | 74·3 ± 12·7 | 79·4 ± 9·9 | 71·9 ± 13·6 | 77·0 ± 11·3 | 73·0 ± 10·6 | 67·8 ± 13·0 | |  |
|  | 3·9 - 7·8 mmol/l [%] | 50·5 ± 13·5 | 51·1 ± 13·0 | 53·8 ± 12·0 | 49·6 ± 14·2 | 52·0 ± 14·9 | 49·0 ± 12·6 | 44·8 ± 12·3 | |  |
|  | >10·0 mmol/l [%] | 19·6 [13·1, 31·3] | 24·1 [14·2, 34·1] | 17·2 [11·8, 25·7] | 29·9 [13·0, 33·7] | 17·9 [13·9, 31·5] | 26·8 [19·0, 33·3] | 29·9 [19·2, 42·4] | |  |
|  | >13·9 mmol/l [%] | 2·4 [1·2, 8·7] | 3·3 [1·4, 7·1] | 2·1 [0·9, 4·9] | 5·7 [1·7, 10·9] | 2·5 [1·4, 5·2] | 5·7 [3·3, 7·0] | 7·9 [3·7, 11·3] | |  |
|  | <3·9 mmol/l [%] | 1·1 [0·2, 1·8] | 1·3 [0·6, 2·2] | 1·2 [0·2, 2·2] | 1·6 [1·0, 2·7] | 1·2 [0·6, 2·8] | 1·3 [0·6, 2·5] | 2·2 [1·0, 2·9] | |  |
|  | <3·0 mmol/l [%] | 0·0 [0·0, 0·2] | 0·1 [0·0, 0·3] | 0·1 [0·0, 0·3] | 0·3 [0·1, 0·4] | 0·2 [0·1, 0·5] | 0·2 [0·1, 0·3] | 0·2 [0·1, 0·5] | |  |
|  | Mean glucose [mmol/L] | 8·4 ± 1·1 | 8·4 ± 1·2 | 8·0 ± 0·9 | 8·6 ± 1·2 | 8·2 ± 1·0 | 8·5 ± 0·9 | 8·9 ± 1·1 | |  |
|  | SD of glucose [mmol/L] | 2·5 [2·2, 3·2] | 2·6 [2·3, 3·2] | 2·4 [2·2, 2·8] | 2·9 [2·3, 3·5] | 2·4 [2·2, 2·8] | 2·8 [2·5, 3·1] | 3·1 [2·6, 3·5] | |  |
|  | CV of glucose [%] | 30·9 [27·5, 34·7] | 32·0 [29·3, 36·1] | 30·4 [27·8, 35·0] | 33·3 [30·9, 37·3] | 31·0 [28·3, 36·4] | 31·6 [30·3, 38·7] | 35·1 [33·5, 37·5] | |  |
|  | Reported burden of carb estimation [0 to 100] | 31·6 ± 24·7 | 43·7 ± 29·1 | 30·9 ± 22·7 | - | - | 39·9 ± 28·4 | - | |  |
|  | Mean absolute CHO estimation error | 23·6 [19·0, 26·2] | 22·8 [19·5, 25·7] | 23·2 [19·5, 24·5] | 22·1 [19·7, 25·2] | - | - | - | |  |
|  | Mean relative CHO estimation error | 0·4 [0·4, 0·5] | 0·4 [0·4, 0·6] | 0·4 [0·4, 0·4] | 0·4 [0·4, 0·5] | - | - | - | |  |
|  | Daily SNAQ use | - | - | 1·6 ± 0·8 | - | - | 1·6 ± 0·9 | - | |  |
|  | Total insulin [IU/day] | 44·9 [39·9, 54·1] | 50·2 [43·9, 69·9] | 43·5 [35·4, 52·2] | 48·4 [38·9, 74·9] | 44·9 [40·2, 54·8] | 48·6 [40·7, 69·9] | 58·7 [41·3, 69·5] | |  |
|  | Manual insulin dose [IU/day] | 16·8 [14·7, 25·8] | 16·7 [13·1, 26·9] | 17·9 [13·9, 25·1] | 22·0 [12·5, 29·7] | 20·7 [14·2, 30·2] | 18·6 [12·6, 28·9] | 16·5 [12·2, 29·1] | |  |
|  | Automated insulin dose [IU/day] | 26·2 [22·6, 31·2] | 36·8 [26·5, 42·9] | 22·5 [20·2, 29·7] | 34·1 [24·1, 41·9] | 24·3 [19·2, 30·7] | 33·9 [27·3, 40·0] | 35·6 [30·4, 43·2] | |  |
|  | Manual insulin frequency [/day) | 5·2 [2·4, 8·0] | 3·9 [0·8, 7·1] | 5·4 [1·5, 9·3] | 4·5 [1·2, 7·8] | 6·7 [3·5, 9·8] | 4·0 [1·3, 6·7] | 4·3 [1·9, 6·7] | |  |
|  | Carbohydrate input [g/day] | 122·2 ± 60·3 | 141·8 ± 67·7 | 134·2 ± 68·9 | 141·6 ± 72·4 | 136·0 ± 66·7 | 132·5 ± 52·3 | 138·2 ± 62·8 | |  |

*Table S8. Data are mean ± SD or median [25th and 75th percentile], CV = coefficient of variation, CHO = carbohydrate. Between-group differences in carbohydrate estimation skills from baseline to the end of the Main Trial Period were assessed using a mixed model with participants as a random effect. Numbers in parentheses refer to the number of participants (n) contributing to the analysis. See Table S3 for further details.*

**Table S9. Sustainability Analysis: Change of glycaemic endpoints and insulin metrics from Pre-SNAQ Period to the Post-SNAQ Period**

|  |  | *Mean change* | *95% CI* | *p-value* |  |
| --- | --- | --- | --- | --- | --- |
|  | **Glucose levels over 24h** |  |  |  |  |
|  | Percentage time with glucose |  |  |  |  |
|  | levels in range at |  |  |  |  |
|  | 3·9 - 10·0mmol/L [%] | -0·5 | -3·4, 2·5 | 0·758 |  |
|  | 3·9 - 7·8 mmol/L [%] | -0·7 | -3·6, 2·3 | 0·655 |  |
|  | >10·0 mmol/L [%] | 0·1 | -2·7, 2·9 | 0·950 |  |
|  | >13·9 mmol/L [%] | -0·4 | -2, 1·1 | 0·582 |  |
|  | <3·9 mmol/L [%] | 0·4 | 0·1, 0·6 | **0**·**005** |  |
|  | <3·0 mmol/L [%] | 0·0 | -0·1, 0·1 | 0·696 |  |
|  | Mean glucose [mmol/L] | -0·1 | -0·3, 0·2 | 0·606 |  |
|  | SD of glucose [mmol/L] | -0·2 | -0·7, 0·3 | 0·489 |  |
|  | CV of glucose [%] | -1·5 | -6, 3 | 0·513 |  |
|  |  |  |  |  |  |
|  | **Postprandial glucose levels** |  |  |  |  |
|  | Percentage time with glucose |  |  |  |  |
|  | levels in range at |  |  |  |  |
|  | 3·9 - 10·0mmol/L [%] | -1·9 | -7·4 , 3·7 | 0·5 |  |
|  | 3·9 - 7·8 mmol/L [%] | -0·3 | -5 , 4·4 | 0·902 |  |
|  | >10·0 mmol/L [%] | -3·5 | -13·8 , 6·7 | 0·486 |  |
|  | >13·9 mmol/L [%]^*^ | 0 |  |  |  |
|  | <3·9 mmol/L [%]^*^ | 0 |  |  |  |
|  | <3·0 mmol/L [%]^*^ | 0 |  |  |  |
|  | Mean glucose [mmol/L] | 0 | -0·5 , 0·4 | 0·942 |  |
|  | SD of glucose [mmol/L] | 0 | -0·1 , 0·2 | 0·85 |  |
|  | CV of glucose [%] | -0·2 | -1·8 , 1·4 | 0·796 |  |
|  | Peak glucose [mmol/L] | 0·1 | -0·9 , 1·2 | 0·776 |  |
|  |  |  |  |  |  |
|  | **Insulin metrics** |  |  |  |  |
|  | Total insulin [IU/day] | 1·0 | -1·2, 3 | 0·394 |  |
|  | Manual insulin [IU/day] | 0·8 | -1·2, 2·8 | 0·425 |  |
|  | Automated insulin [IU/day] | 0·6 | -3·5, 4·7 | 0·783 |  |
|  | Manual bolus frequency [/day) | 0·4 | -0·4, 1·3 | 0·322 |  |
|  | Carbohydrate input [g/day] | 7·7 | -6·2, 21·6 | 0·283 |  |

*Table S9. Sustainability Analysis: Change of glycaemic endpoints and insulin metrics from Pre-SNAQ Period to the Post-SNAQ Period, CV = coefficient of variation.* * *Due to the lack of variability in the data, with most participants spending no time in the respective range, no statistical testing was performed.*

**Table S10. Results from the app satisfaction survey**

|  | The application simplified my nutrition plan | Absolute (n = 41) | Percentage [%] |  |
| --- | --- | --- | --- | --- |
|  | I strongly disagree | 4 | 9·8 |  |
|  | I disagree | 7 | 17·1 |  |
|  | I am neutral | 21 | 51·2 |  |
|  | I agree | 8 | 19·5 |  |
|  | I strongly agree | 1 | 2·4 |  |
|  |  |  |  |  |
|  | I used the app to help with carbohydrate estimation at least twice a day | Absolute (n = 41) | Percentage [%] |  |
|  | I strongly disagree | 1 | 2·4 |  |
|  | I disagree | 5 | 12·2 |  |
|  | I am neutral | 4 | 9·8 |  |
|  | I agree | 19 | 46·3 |  |
|  | I strongly agree | 12 | 29·3 |  |
|  |  |  |  |  |
|  | The app improved my blood glucose control (more time in target range or fewer correction boluses) | Absolute (n = 41) | Percentage [%] |  |
|  | I strongly disagree | 8 | 19·5 |  |
|  | I disagree | 7 | 17·1 |  |
|  | I am neutral | 16 | 39 |  |
|  | I agree | 8 | 19·5 |  |
|  | I strongly agree | 2 | 4·9 |  |
|  |  |  |  |  |
|  | The app has helped me to refresh/improve my knowledge about nutrition | Absolute (n = 41) | Percentage [%] |  |
|  | I strongly disagree | 4 | 9·8 |  |
|  | I disagree | 7 | 17·1 |  |
|  | I am neutral | 6 | 14·6 |  |
|  | I agree | 21 | 51·2 |  |
|  | I strongly agree | 3 | 7·3 |  |
|  |  |  |  |  |
|  | The app has led to changes in my food choices or behavior | Absolute (n = 41) | Percentage [%] |  |
|  | I strongly disagree | 10 | 24·4 |  |
|  | I disagree | 18 | 43·9 |  |
|  | I am neutral | 8 | 19·5 |  |
|  | I agree | 5 | 12·2 |  |
|  | I strongly agree | 0 | 0 |  |
|  |  |  |  |  |
|  | I am satisfied with the design of the app's user interface | Absolute (n = 41) | Percentage [%] |  |
|  | I strongly disagree | 1 | 2·4 |  |
|  | I disagree | 0 | 0 |  |
|  | I am neutral | 7 | 17·1 |  |
|  | I agree | 25 | 61 |  |
|  | I strongly agree | 8 | 19·5 |  |
|  |  |  |  |  |
|  | I am satisfied with the overall performance of the app | Absolute (n = 41) | Percentage [%] |  |
|  | I strongly disagree | 1 | 2·4 |  |
|  | I disagree | 16 | 39 |  |
|  | I am neutral | 12 | 29·3 |  |
|  | I agree | 11 | 26·8 |  |
|  | I strongly agree | 1 | 2·4 |  |
|  |  |  |  |  |
|  | The app recognized the food category correctly in the majority of cases | Absolute (n = 41) | Percentage [%] |  |
|  | I strongly disagree | 4 | 9·8 |  |
|  | I disagree | 15 | 36·6 |  |
|  | I am neutral | 6 | 14·6 |  |
|  | I agree | 15 | 36·6 |  |
|  | I strongly agree | 1 | 2·4 |  |
|  |  |  |  |  |
|  | The app correctly estimated the quantity of the meal in most cases | Absolute (n = 41) | Percentage [%] |  |
|  | I strongly disagree | 5 | 12·2 |  |
|  | I disagree | 14 | 34·1 |  |
|  | I am neutral | 10 | 24·4 |  |
|  | I agree | 11 | 26·8 |  |
|  | I strongly agree | 1 | 2·4 |  |
|  |  |  |  |  |
|  | The app overestimated the amount of the meal | Absolute (n = 41) | Percentage [%] |  |
|  | I strongly disagree | 1 | 2·4 |  |
|  | I disagree | 11 | 26·8 |  |
|  | I am neutral | 10 | 24·4 |  |
|  | I agree | 16 | 39 |  |
|  | I strongly agree | 3 | 7·3 |  |
|  |  |  |  |  |
|  | The app underestimated the quantity of the meal | Absolute (n = 41) | Percentage [%] |  |
|  | I strongly disagree | 3 | 7·3 |  |
|  | I disagree | 15 | 36·6 |  |
|  | I am neutral | 12 | 29·3 |  |
|  | I agree | 11 | 26·8 |  |
|  | I strongly agree | 0 | 0 |  |
|  |  |  |  |  |
|  | The app estimated the amount of the meal without a consistent pattern | Absolute (n = 41) | Percentage [%] |  |
|  | I strongly disagree | 2 | 4·9 |  |
|  | I disagree | 8 | 19·5 |  |
|  | I am neutral | 14 | 34·1 |  |
|  | I agree | 16 | 39 |  |
|  | I strongly agree | 1 | 2·4 |  |
|  |  |  |  |  |
|  | Would you continue using the application after the end of the study? | Absolute (n = 41) | Percentage [%] |  |
|  | I strongly disagree | 5 | 12·2 |  |
|  | I disagree | 9 | 22 |  |
|  | I am neutral | 11 | 26·8 |  |
|  | I agree | 14 | 34·1 |  |
|  | I strongly agree | 2 | 4·9 |  |
|  |  |  |  |  |
|  | Would you recommend the app to other persons? | Absolute (n = 41) | Percentage [%] |  |
|  | I strongly disagree | 3 | 7·3 |  |
|  | I disagree | 8 | 19·5 |  |
|  | I am neutral | 11 | 26·8 |  |
|  | I agree | 16 | 39 |  |
|  | I strongly agree | 3 | 7·3 |  |
|  |  |  |  |  |
|  | I would like to use the app more frequently | Absolute (n = 41) | Percentage [%] |  |
|  | I strongly disagree | 6 | 14·6 |  |
|  | I disagree | 4 | 9·8 |  |
|  | I am neutral | 14 | 34·1 |  |
|  | I agree | 16 | 39 |  |
|  | I strongly agree | 1 | 2·4 |  |
|  |  |  |  |  |
|  | I experienced the app as unnecessary complex | Absolute (n = 41) | Percentage [%] |  |
|  | I strongly disagree | 11 | 26·8 |  |
|  | I disagree | 19 | 46·3 |  |
|  | I am neutral | 6 | 14·6 |  |
|  | I agree | 5 | 12·2 |  |
|  | I strongly agree | 0 | 0 |  |
|  |  |  |  |  |
|  | I think the app is easy to use | Absolute (n = 41) | Percentage [%] |  |
|  | I strongly disagree | 0 | 0 |  |
|  | I disagree | 2 | 4·9 |  |
|  | I am neutral | 3 | 7·3 |  |
|  | I agree | 25 | 61 |  |
|  | I strongly agree | 11 | 26·8 |  |
|  |  |  |  |  |
|  | I think I would need the support of a person with a technical background to use the app | Absolute (n = 41) | Percentage [%] |  |
|  | I strongly disagree | 24 | 58·5 |  |
|  | I disagree | 16 | 39 |  |
|  | I am neutral | 1 | 2·4 |  |
|  | I agree | 0 | 0 |  |
|  | I strongly agree | 0 | 0 |  |
|  |  |  |  |  |
|  | I found the various features in this app to be well integrated (I thought that the different features worked well together) | Absolute (n = 41) | Percentage [%] |  |
|  | I strongly disagree | 1 | 2·4 |  |
|  | I disagree | 6 | 14·6 |  |
|  | I am neutral | 8 | 19·5 |  |
|  | I agree | 21 | 51·2 |  |
|  | I strongly agree | 5 | 12·2 |  |
|  |  |  |  |  |
|  | I found that this app contained too many inconsistencies (I thought that the different features were not well coordinated) | Absolute (n = 41) | Percentage [%] |  |
|  | I strongly disagree | 5 | 12·2 |  |
|  | I disagree | 18 | 43·9 |  |
|  | I am neutral | 12 | 29·3 |  |
|  | I agree | 4 | 9·8 |  |
|  | I strongly agree | 2 | 4·9 |  |
|  |  |  |  |  |
|  | I can imagine that most people would learn to use this app very quickly | Absolute (n = 41) | Percentage [%] |  |
|  | I strongly disagree | 0 | 0 |  |
|  | I disagree | 2 | 4·9 |  |
|  | I am neutral | 3 | 7·3 |  |
|  | I agree | 25 | 61 |  |
|  | I strongly agree | 11 | 26·8 |  |
|  |  |  |  |  |
|  | I found the app to be very cumbersome to use | Absolute (n = 41) | Percentage [%] |  |
|  | I strongly disagree | 11 | 26·8 |  |
|  | I disagree | 18 | 43·9 |  |
|  | I am neutral | 5 | 12·2 |  |
|  | I agree | 7 | 17·1 |  |
|  | I strongly agree | 0 | 0 |  |
|  |  |  |  |  |
|  | I felt safe using the app | Absolute (n = 41) | Percentage [%] |  |
|  | I strongly disagree | 1 | 2·4 |  |
|  | I disagree | 6 | 14·6 |  |
|  | I am neutral | 3 | 7·3 |  |
|  | I agree | 19 | 46·3 |  |
|  | I strongly agree | 12 | 29·3 |  |
|  |  |  |  |  |
|  | I had to learn a lot before I could use this app | Absolute (n = 41) | Percentage [%] |  |
|  | I strongly disagree | 21 | 51·2 |  |
|  | I disagree | 16 | 39 |  |
|  | I am neutral | 1 | 2·4 |  |
|  | I agree | 3 | 7·3 |  |
|  | I strongly agree | 0 | 0 |  |
|  |  |  |  |  |
|  | To what extent did you feel fatigued by estimating carbohydrates while using the app? | Absolute (n = 41) | Percentage [%] |  |
|  | Less than before | 7 | 17·5 |  |
|  | Same as before | 27 | 67·5 |  |
|  | More than before | 6 | 15 |  |
|  |  |  |  |  |
|  | How concerned were you while using the app that an inaccurate estimation of carbohydrates could lead to a dangerous event? | Absolute (n = 41) | Percentage [%] |  |
|  | Less than before | 2 | 5 |  |
|  | Same as before | 26 | 65 |  |
|  | More than before | 12 | 30 |  |
|  |  |  |  |  |
|  | How concerned were you while using the app that an inaccurate estimation of carbohydrates could lead to long-term complications? | Absolute (n = 41) | Percentage [%] |  |
|  | Less than before | 3 | 7·5 |  |
|  | Same as before | 33 | 82·5 |  |
|  | More than before | 4 | 10 |  |
|  |  |  |  |  |
|  | To what extent did you feel that estimating carbohydrates while using the app negatively affected your social interactions? | Absolute (n = 41) | Percentage [%] |  |
|  | Less than before | 3 | 7·5 |  |
|  | Same as before | 29 | 72·5 |  |
|  | More than before | 8 | 20 |  |
|  |  |  |  |  |
|  | To what extent do you feel that the limited selection of foods and the difficulty in estimating carbohydrates have restricted your social life? | Absolute (n = 41) | Percentage [%] |  |
|  | Not at all | 19 | 47·5 |  |
|  | A little | 18 | 45 |  |
|  | Much | 3 | 7·5 |  |
|  | Very much | 0 | 0 |  |
|  |  |  |  |  |

*Table S10. Results from the app satisfaction survey, which was filled by all participants after 3 weeks of SNAQ use. Numbers in parentheses refer to the number of participants (n) contributing to the analysis. See Table S3 for further details.*

**Table S11. Safety endpoints**

|  |  | **Main Trial Period** | | **Post-SNAQ Period** | **SNAQ Period** | **Post-SNAQ Period** | **Follow-up Period** | |  |
| --- | --- | --- | --- | --- | --- | --- | --- | --- | --- |
|  |  | Intervention (n = 22) | Control (n = 22) | Intervention (n = 21) | Control (n = 20) | Control (n = 20) | Intervention (n = 20) | Control (n = 20) |  |
|  |  | SNAQ | Usual care | SNAQ off | SNAQ | Usual care | Usual care | Usual care |  |
|  | **Serious adverse events, n** | 0 | 0 | 0 | 0 | 0 | 1^†^ | 0 |  |
|  | **Other adverse events, n** | 0 | 0 | 0 | 0 | 1* | 0 | 0 |  |

*Table S11. Safety endpoints: Numbers in parentheses refer to active participants (n) in the corresponding study period. See Table S3 for further details. * hypoglycemia grade II,* † *pleural and pericardial effusion*

**Table S12. Overview of postprandial glucose metrics (calculated over 3 hours-post meal) according absolute deviations (in grams) between SNAQ-generated and participant-entered carbohydrate estimates**

|  | **> 20g** | **20 to 10g** | **10 to 5g** | **5 to 0g** | **Identical** | **0 to 5g** | **5 to 10g** | **10 to 20g** | **>20g** | **No SNAQ** | **P-value** | |  |
| --- | --- | --- | --- | --- | --- | --- | --- | --- | --- | --- | --- | --- | --- |
|  | *less carbohydrates than suggested by SNAQ entered into the AID system* | | | |  | *more carbohydrates than suggested by SNAQ entered into the AID system* | | | |  |  | |  |
| **Frequency** | 19·7% | 8·2% | 7·7% | 11·6% | 19·2% | 10% | 5·2% | 7·6% | 10·7% | 61·4% |  | |  |
| **Percent of time with sensor glucose level:** | | | | | | | | | | | | | |
| 3·9-10·0 mmol/L [%] | 71·5 ± 29·1 | 76·9 ± 30·3 | 73·8 ± 29·3 | 73·6 ± 29·4 | 61·8 ± 36·2 | 80·7 ± 26·2 | 73·2 ± 31·1 | 67 ± 33·7 | 72·6 ± 32·1 | 62·8 ± 34·3 | **p = <0·001** | |  |
| 3·9-7·8 mmol/L [%] | 44·8 ± 32·5 | 52·7 ± 30·6 | 46·7 ± 33 | 44·3 ± 31·7 | 38·1 ± 33·9 | 51·6 ± 31·4 | 38·4 ± 29·4 | 42·6 ± 36 | 47·9 ± 35 | 38·2 ± 33 | **p = <0·001** | |  |
| >10·0 mmol/L [%] | 14·9 [0, 50·8] | 0 [0, 23·2] | 11·3 [0, 43·9] | 3·6 [0, 43] | 24 [0, 67·4] | 0 [0, 35·9] | 0 [0, 47] | 18·8 [0, 60·9] | 0 [0, 47·5] | 23·8 [0, 61·9] | **p = <0·001** | |  |
| >13·9 mmol/L [%] | 0 [0, 0] | 0 [0, 0] | 0 [0, 0] | 0 [0, 0] | 0 [0, 6·2] | 0 [0, 0] | 0 [0, 0] | 0 [0, 0] | 0 [0, 0] | 0 [0, 2·8] | **p = <0·001** | |  |
| <3·9 mmol/L [%] | 0 [0, 0] | 0 [0, 0] | 0 [0, 0] | 0 [0, 1·2] | 0 [0, 0] | 0 [0, 0] | 0 [0, 0] | 0 [0, 0] | 0 [0, 0] | 0 [0, 0] | **p = 0·032** | |  |
| <3·0 mmol/L [%] | 0 [0, 0] | 0 [0, 0] | 0 [0, 0] | 0 [0, 0] | 0 [0, 0] | 0 [0, 0] | 0 [0, 0] | 0 [0, 0] | 0 [0, 0] | 0 [0, 0] | p = 0·955 | |  |
| **Mean glucose [mmol/L]** | 8·4 ± 2·1 | 7·8 ± 2·4 | 8·3 ± 1·9 | 8·2 ± 2·2 | 9·4 ± 3·3 | 8 ± 2·2 | 8·8 ± 2·9 | 8·5 ± 2·4 | 8·5 ± 2·7 | 9·2 ± 2·9 | **p = <0·001** | |  |
| **SD of glucose [mmol/L]** | 1·5 [1, 2·2] | 1·4 [1, 1·7] | 1·5 [1, 2] | 1·4 [0·9, 1·8] | 1·5 [0·9, 2·1] | 1·4 [0·8, 1·7] | 1·4 [1, 1·8] | 1·4 [0·9, 2] | 1·4 [0·9, 2·1] | 1·5 [1, 2·2] | **p = <0·001** | |  |
| **CV of glucose[%]** | 17·3 [13·4, 27·9] | 19·2 [13·6, 25·1] | 18·6 [13, 23·3] | 18·2 [12·2, 22·8] | 15·8 [11·2, 24] | 16·4 [11·1, 22·5] | 16·3 [9·5, 21·3] | 16·6 [10·9, 25·7] | 17·2 [12·2, 23·3] | 17·9 [12, 25] | p = 0·4502 | |  |
| **Peak glucose [mmol/L]** | 10·8 [9, 12·8] | 9·8 [8·4, 12] | 10·4 [9·1, 12·6] | 10·1 [8·8, 12·4] | 11·5 [8·9, 14] | 9·9 [8·5, 11·9] | 10 [8·8, 12·3] | 10·7 [8·6, 12·7] | 10 [8·6, 12·8] | 11·6 [9·4, 14] | **p = <0·001** |  |  |

*Table S12. Data are mean ± SD or median [25th and 75th percentile], CV = coefficient of variation. Data generated across all participants during periods with requested SNAQ use. AID system = automated insulin delivery system. The p-value is calculated for the overall effect of agreement category (i.e., deviation between carbohydrate estimates provided by SNAQ and those entered into the AID system) using a Wald chi-square test.*

**Other Supplementary Material**

**Carbohydrate estimation quiz**


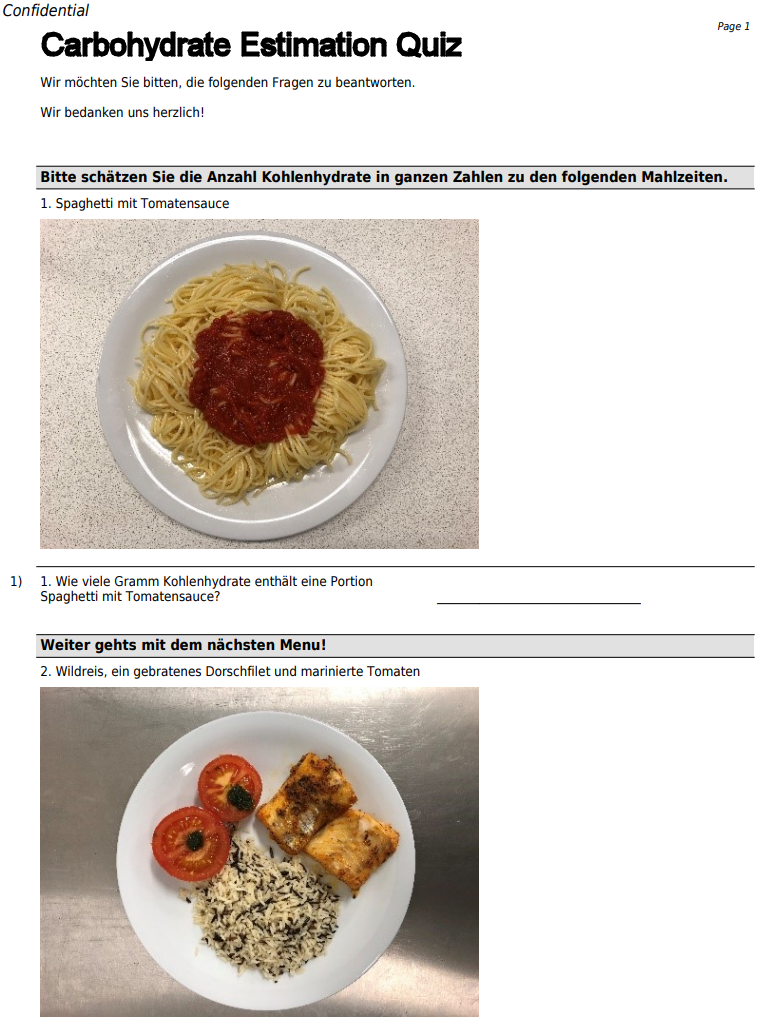


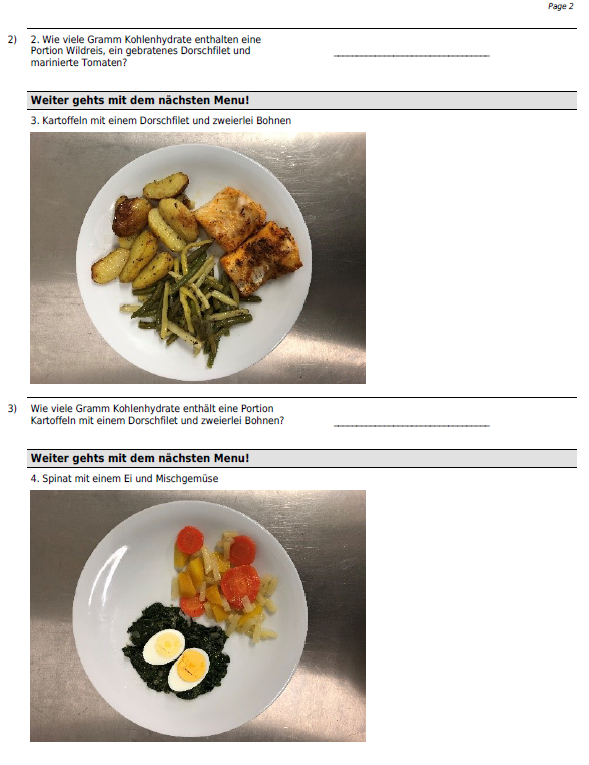


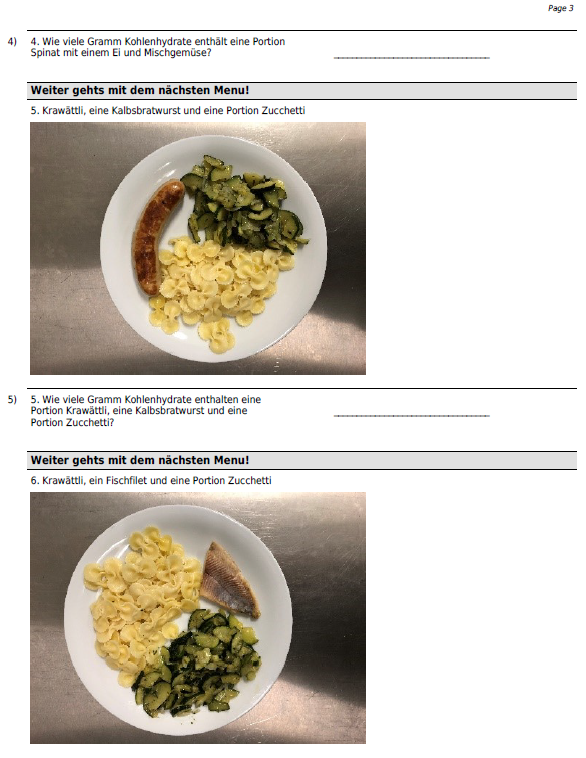


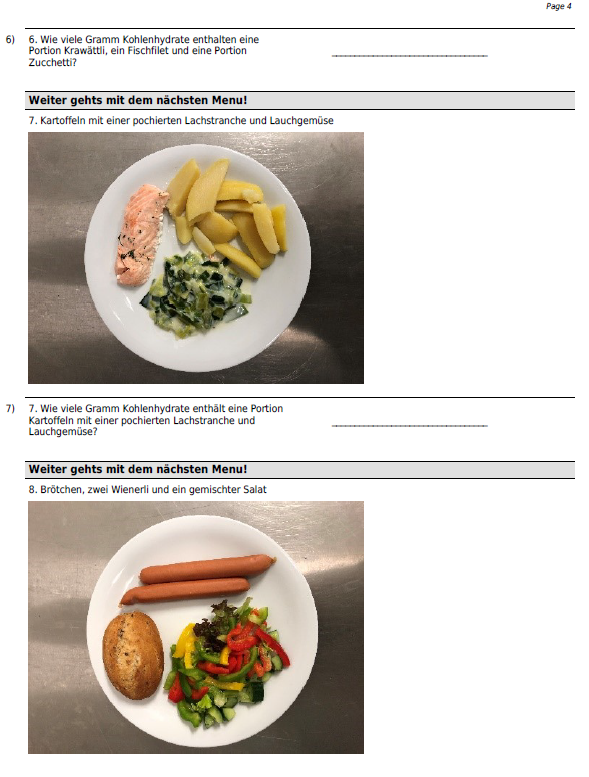


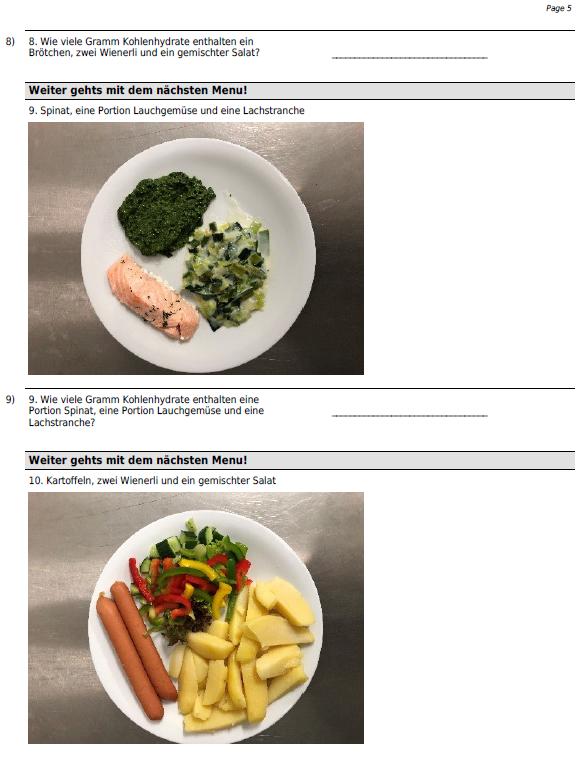


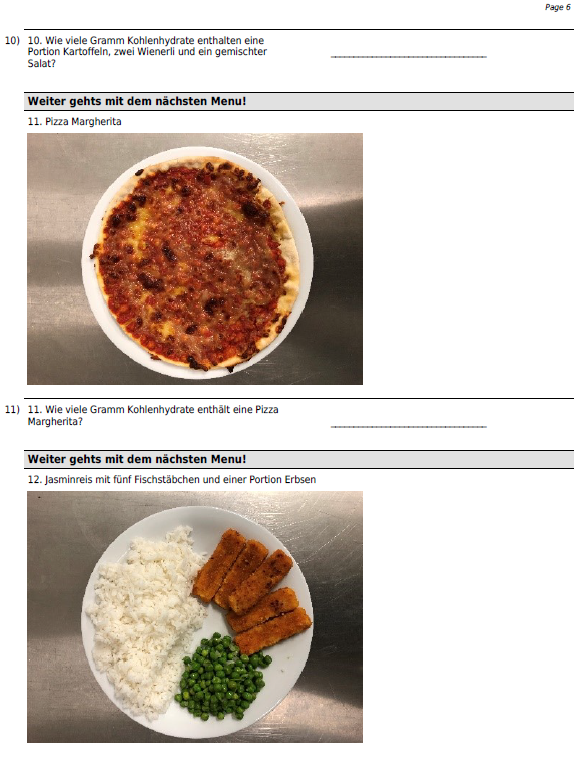


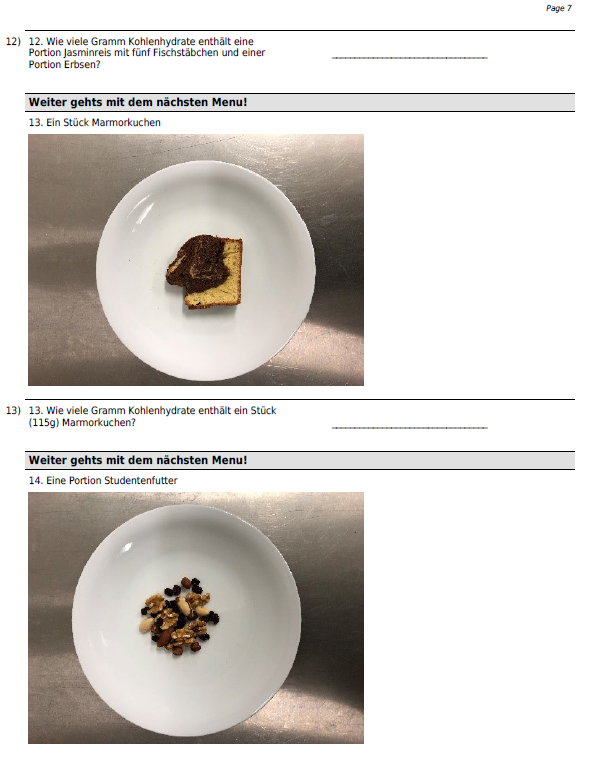


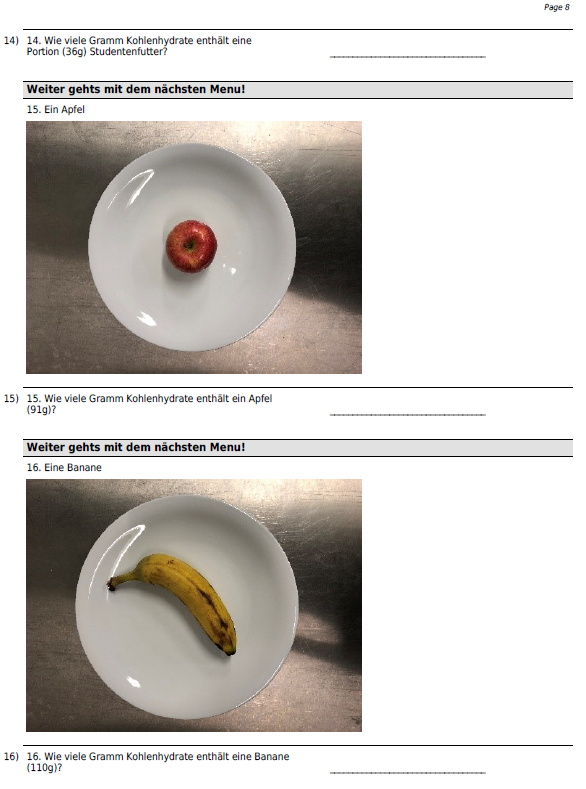


*Carbohydrate estimation quiz with 16 randomly selected meals, each containing between 7 and 140 grams of carbohydrates. The carbohydrate estimation quiz was done by each participant at baseline and after the Main Trial Period.*
