# Supplementary material for: App-based automated meal analysis in adults with type 1 diabetes using automated insulin delivery: a randomized controlled trial
Source: eClinicalMedicine. 2025 Oct 8;89:103537. doi: 10.1016/j.eclinm.2025.103537 (PMC12538901; doi:10.1016/j.eclinm.2025.103537)
Supplement: Protocol_SUMMIT_V1_signed [file mmc2.pdf]

Supporting Meal Management in Type 1 Diabetes (SUMMIT1)

Study Type:	Other Clinical Trial according to ClinO, Chapter 4
Risk Categorisation:	Risk category A according to ClinO, Art. 61 (minimal risks and burdens)
Study Registration:	Clinicialtrials.gov: NCT number pending<br>Swiss National Clinical Trial Portal: SNCTP number pending
Sponsor-Investigator:	Prof Lia Bally MD PhD<br>Department of Diabetes, Endocrinology, Nutritional Medicine and Metabolism<br>Inselspital Bern, University Hospital Bern and University of Bern<br>Freiburgstrasse 15, Bern<br>Switzerland
Investigated Intervention:	Meal management aid
Protocol ID	SUMMIT1
Version and Date:	Version 1.0 (dated 07/12/2022)

CONFIDENTIALITY STATEMENT

The information contained in this document is confidential and the property of the sponsor. The information may not - in full or in part - be transmitted, reproduced, published, or disclosed to others than the applicable Competent Ethics Committee(s) and Regulatory Authority(ies) without prior written authorisation from the sponsor except to the extent necessary to obtain informed consent from those who will participate in the study.

PROTOCOL SIGNATURE FORM

Study Title	Supporting Meal Management in Type 1 Diabetes
Study ID	SUMMIT1

The Sponsor-Investigator has approved the protocol version 1.0 (dated 07/12/2022) and confirm hereby to conduct the study according to the protocol, current version of the World Medical Association Declaration of Helsinki, and ICH-GCP guidelines as well as the local legally applicable requirements.

Sponsor-Investigator:

Name: *Prof Lia Bally*

Date: 07.12.2022

Signature: ______________________

TABLE OF CONTENTS

TABLE OF CONTENTS 3
GLOSSARY OF ABBREVATIONS 4
1 BACKGROUND AND RATIONALE 5
2 STUDY OBJECTIVES AND DESIGN 6
2.1 Hypothesis and primary objective 6
2.2 Study design 8
2.3 Study intervention 8
3 STUDY POPULATION AND STUDY PROCEDURES 9
3.1 Inclusion and exclusion criteria, justification of study population 9
3.2 Recruitment, screening and informed consent procedure 9
3.3 Study procedures 10
3.4 Withdrawal and discontinuation 13
3.5 Randomization 13
4 STATISTICS AND METHODOLOGY 13
4.1. Statistical analysis plan and sample size calculation 13
4.2. Handling of missing data and drop-outs 14
5 REGULATORY ASPECTS AND SAFETY 14
5.1 Local regulations / Declaration of Helsinki 14
5.2 (Serious) Adverse Events and notification of safety and protective measures 14
5.3 (Periodic) safety reporting 16
5.4 Pregnancy 16
5.5 Amendments 16
5.6 Notification and reporting upon completion, discontinuation or interruption of the study 16
5.7 Insurance 16
6 FURTHER ASPECTS 17
6.1 Overall ethical considerations 17
6.2 Risk-benefit assessment 17
7 QUALITY CONTROL AND DATA PROTECTION 17
7.1 Quality measures 17
7.2 Data recording and source data 18
7.3 Confidentiality and coding 18
7.4 Retention of study data 19
8 MONITORING AND REGISTRATION 19
9. FUNDING / PUBLICATION / DECLARATION OF INTEREST 20
10. REFERENCES 21
Appendix 1: Schedule of assessments and procedures 22

GLOSSARY OF ABBREVATIONS

AE	*Adverse Event*
ASR	*Annual Safety Repot*
BASEC	*Business Administration System for Ethical Committees*
CGM	*Continuous Glucose Monitor*
CHO	*Carbohydrates*
ClinoO	*Ordinance on Clinical Trials in Human Research (in German: KlinV, in French: OClin, in Italian: OSRUm)*
CRF	*Case Report Form*
CTCAE	*Common Terminology Criteria for Adverse Events*
CV	*Coefficients of Variation*
DD	*Device Deficiency*
EC	*Ethics Committee*
eCRF	*electronic Case Report Form*
EFSD	*European Foundation for the Study of Diabetes*
FADP	*Federal Act on Data Protection (in German: DSG, in French: LPD, in Italian: LPD)*
FOPH	*Federal Office of Public Health*
GCP	*Good Clinical Practice*
GLM	*General Linear Models*
HRA	*Human Research Act (in German: HFG, in French: LRH, in Italian: LRUm)*
ICH	*International Conference on Harmonisation*
SAE	*Serious Adverse Event*
SCTO	*the Swiss Clinical Trial organization*
SD	*Standard Deviation*
SNCTP	*Swiss National Clinical Trial Portal*
SSL	*Secure Sockets Layer*
WHO	*World Health Organization*

1 BACKGROUND AND RATIONALE

Diet and physical activity are critically important in the lifestyle of people with type 1 diabetes. When diagnosed with the disease, people with type 1 diabetes are educated about nutritional goals and how to estimate nutritional content of food. Carbohydrates are the food component with the greatest impact on blood glucose levels and typical sources in the diet include starches, some vegetables, fruits, dairy products and sugars [1]. Thus, people with type 1 diabetes are primarily being trained to estimate the carbohydrate content of food, a task that is also referred to as carbohydrate counting. Different methods can be used to count carbohydrate in food and drink. These include reading the nutritional labels, consulting reference books or websites, carrying a database on a personal digital assistant or using exchange tables which provides the carbohydrate content for typical serving sizes (e.g. 1 slice of bread). While nutritional information can be accessed through the above mentioned methods, the quantification of the portion sizes (if not indicated on the food package) requires the additional use of scale or measuring vessel. Given the required effort and time investment related to these methods, the great majority of people with type 1 diabetes count carbohydrates by visual estimation and experience. As a consequence, people's estimate often deviate substantially from ground truth values and average carbohydrate estimation errors reported in the literature are 20% or higher [2].

Of note, more than 60% of individuals with diabetes report having trouble with carbohydrate counting, despite their awareness on its importance [3]. Even in patients who are confident in applying carbohydrate counting, the daily task is perceived as major burden of diabetes self-management [4].

Since carbohydrate counting is particularly demanding when eating fresh, non-packaged foods, a concerning trend towards unhealthy dietary choices with preference of prepackaged foods (with accessible nutrition facts) over whole foods is increasingly observed in people with type 1 diabetes [5, 6]. This is paralleled by an increasing prevalence of overweight and obesity in the type 1 diabetes population [7].

Thus, even with the latest hybrid closed-loop insulin delivery technologies, adequate nutrition knowledge remains a cornerstone for satisfactory glucose control, metabolic health, and prevention of diabetes-related complications and comorbidities [8].

With the development of new technologies embedded in modern smartphones (i.e. depth sensors), image-based methods to support food assessment have become widely available. Of particular use is the employment of well-established computer vision methodologies to estimate the quantity of food. When combined with food-recognition technologies and information from nutritional databases, a proposition of the nutritional content (e.g. carbohydrates, fat, proteins, fibres) can be made to the user on the basis of captured images and obviates the need for error prone visual estimations and mental calculations. Several such applications have become available and can support monitoring the diet as part of lifestyle management.

Insights from a recent online survey suggest that a high proportion of people with type 1 diabetes believe that such new technologies for meal management could facilitate their daily self-management and would be interested in using such technology. Moreover, according to a recent study, such digital tools may promote diabetes education and food literacy which may particularly benefit those with a lower education level and with a history of depression [4].

Amongst several options (e.g. Foodvisor, Calorie-Mamma, Lifesum) for image-based food tracking and analysis, SNAQ is one of the most commonly used app in people with type 1 diabetes. Up to date, more than 40000 users have downloaded the SNAQ app in their phones, of which 2,500 are living in Switzerland.

We have previously demonstrated that the system estimates the macronutrient content of real meals with satisfying accuracy [9].

However, evidence with regards to the effect of the food analysis on daily self-management of people with type 1 diabetes (e.g. glucose control, meal patterns, perceived benefits) is currently lacking. We therefore aim to address these aspects in a randomized-controlled study contrasting the use of the SNAQ app with people's traditional meal management techniques.

2 STUDY OBJECTIVES AND DESIGN

2.1 Hypothesis and primary objective

Hypothesis
The null hypothesis that there is no difference in the true mean time spent in the target glucose range (3.9 to 10.0mmol/L), will be tested against the alternative hypothesis that the use of the food analysis app, SNAQ, for meal management is superior to traditional meal management (two-sided alternative).

Primary objective
The primary objective is to assess the efficacy of the food analysis app, SNAQ, to improve glucose control in people with type 1 diabetes.

Further objectives
Further objectives are to assess the sustainability of potential improvements, the usability of the food analysis app, SNAQ, as well as its impact on nutrition literacy.

Primary endpoint:

The primary objective will be addressed by evaluating the following endpoint:

1) Percentage of time with sensor glucose in the target range (3.9 to 10.0mmol/L, %)

Note: Time in target range will be calculated over the complete 3-week intervention period (Day 1 to Day 21).

Secondary endpoints:

The following endpoints will be assessed to evaluate the effect on glucose control:

2) Percentage of time with sensor glucose in hyperglycaemia (>10.0mmol/L, %)

Note: Time in hyperglycaemia during the intervention period will be calculated over the 3-week intervention period (Day 1 to Day 21).

3) Percentage of time with sensor glucose in hypoglycaemia (<3.9mmol/L, %)

Note: Time in hyperglycaemia during the intervention period will be calculated over the 3-week intervention period (Day 1 to Day 21).

The following endpoints will be assessed to evaluate the effect on postprandial glucose control:

4) Percentage of postprandial time with sensor glucose in target range (3.9 to 10.0mmol/L, %).

Note: Time in target range during the intervention period will be calculated over the 3-week intervention period (Day 1 to Day 21). Postprandial periods are defined as the 180 min following main meal intake. Main meals are defined as CHO amounts above 25g entered by the participants in the hybrid closed-loop system.

5) Percentage of postprandial time with sensor glucose in hyperglycaemia (>10.0mmol/L, %).

Note: Time in hyperglycaemia during the intervention period will be calculated over the 3-week intervention period (Day 1 to Day 21). Postprandial periods are defined as the 180 min following main meal intake. Main meals are defined as CHO amounts above 25g entered by the participants in the closed-loop system.

6) Percentage of postprandial time with sensor glucose in hypoglycaemia (<3.9mmol/L, %).

Note: Time in hypoglycaemia during the intervention period will be calculated over the 3-week intervention period (Day 1 to Day 21). Postprandial periods are defined as the 180 min following main meal intake. Main meals are defined as CHO amounts above 25g entered by the participants in the hybrid closed-loop system.

The following endpoints will be assessed to evaluate the sustainability of potential improvements:

7) Change in the percentage of time with sensor glucose in target range (3.9 to 10.0mmol/L, %)
8) Change in the percentage of time with sensor glucose in hyperglycaemia (>10.0mmol/L, %)
9) Change in the percentage of time with sensor glucose in hypoglycaemia (<3.9mmol/L, %)
10) Change in the percentage of time with postprandial sensor glucose in target range (3.9 to 10.0mmol/L, %).
11) Change in the percentage of time with postprandial sensor glucose in hyperglycaemia (>10.0mmol/L, %).
12) Change in the percentage of postprandial time with sensor glucose in hypoglycaemia (<3.9mmol/L, %).

Note: For all outcomes evaluating sustainability of the improvements, glucose control endpoints calculated at at baseline and in the 3 weeks period of SNAQ use will be compared with the variables calculated during the 3 weeks following discontinuation of the app. Baseline glucose control endpoints will be calculated over the 14 days prior to randomization (day -14 to day -1). Glucose control endpoints during the 3 weeks following discontinued use of the SNAQ app will be calculated as follow: Visit 2 + 1 Day to Visit 2 + 21 Days for the intervention group and Visit 3 + 1 Day to Visit 3 + 21 Days for the control group. Postprandial periods are defined as above.

Pre-defined exploratory endpoints:
The following endpoints will be examined to assess various aspects of SNAQ app use in terms of usefulness, nutrition literacy, psychosocial, and behavioral impact. Pre-specified exploratory endpoints will be reported as point estimates with 95% confidence intervals (unadjusted for multiple testing), so definite inferences cannot be made regarding those endpoints.

Usability and patient reported outcomes:
- Engagement with the SNAQ app in the 3-week follow-up period (frequency of use)
- Technology satisfaction (assessed using a self-designed feedback questionnaire) and psychometric questionnaires

Nutrition literacy
- Carbohydrate estimation skills (absolute and relative errors from the carbohydrate quiz)

Meal management behavior
- Meal patterns (Timing of meals, macronutrient content of meals)
- Daily number of manual boluses,
- Total daily Insulin dose (basal dose, bolus dose and total dose)

Glucose control
In addition to the above mentioned primary and secondary outcomes, several further metrics of glucose control will be analysed, including the following: % time 10.1-13.9 mmol/L, %time >13.9 mmol/L, % time 3.0-3.9 mmol/L, %time <3.0 mmol/L, mean glucose, Standard Deviation-SD and Coefficients of Variation-CV of sensor glucose).

2.2 Study design

Main study
The study will follow a randomized two-arm parallel design. Study visits will be done remotely via video calls or in-clinic when coinciding with usual care appointments. Following a baseline visit and before randomization, baseline characteristics and medical history of the participants will be collected (as detailed in section 4.3). Following randomization, the intervention group will use SNAQ app for the first 3 weeks while the control group will proceed without any modification/intervention by the study team. After the first 3 weeks, the control group will undergo 3 weeks of SNAQ app use (weeks 4-6). At the end of their respective SNAQ app periods (weeks 4-6 for the intervention group and weeks 7-9 for the control group), both groups will discontinue the use of SNAQ app for 3 weeks to assess sustainability of potential effects. Finally, both groups will be offered to use SNAQ app for 3 additional weeks as per their preference (follow-up period). The study design is illustrated in **Figure 1**.

2.3 Study intervention

SNAQ is a smartphone food analysis app that estimates the macronutrient content of a meal, based on a single image. The app first determines meal content in terms of food components with input from the user to correct or add further components (e.g. foods, ingredients, sauces, herbs or seasonings). Then, the total macronutrient and energy content of the meal is determined based on the estimated volume and information from a nutritional database. Of note, the application also allows for assessing nutritional content of packaged foods by means of a barcode scanning function. The user can always adapt proposed nutritional contents at their own discretion. Meal macronutrients alongside the food pictures are collected in a detailed log which allows users to review their dietary choices. The product is not conceived by its manufacturer to be used for medical purposes and can thus not be considered a medical device.

3 STUDY POPULATION AND STUDY PROCEDURES

3.1 Inclusion and exclusion criteria, justification of study population

Subjects fulfilling all of the following inclusion criteria are eligible for the investigation:

- Written informed consent
- Adults (aged 18 years or older)
- Type 1 diabetes (as defined by World Health Organization-WHO for at least 12 month)
- Current use of a commercial hybrid closed-loop system
- HbA1c≤12% (measured within the past 3 months)
- Willing to use the SNAQ app on a daily basis for over 3 weeks
- The participant is willing to follow study specific instructions and share their treatment data with the study team

The presence of any of the following exclusion criteria will lead to the exclusion of the subject

- Any physical or psychological disease or condition likely to interfere with the normal conduct of the study and interpretation of the study results
- Previous use of SNAQ app for more than 5 days within the past 3 months
- Self-reported pregnancy, planed pregnancy within next 3 months or breast-feeding
- Severe visual impairment
- Severe hearing impairment
- Lack of reliable telephone facility for contact
- Concomitant participation in another trial that interferes with the normal conduct of the study and interpretation of the study results
- Participant not proficient in German

Comment:

1) *The exclusion of pregnant and breastfeeding participants is not due to potential safety issues but due to concerns regarding interpretation of study results (impact of pregnancy on physiology, psychometrics and behaviour patterns).*
2) *If the last measurement of HbA1c levels is older than 3 months, a new measurement of HbA1c will be performed if the participant consents to that, if not, the participant will be excluded.*

3.2 Recruitment, screening and informed consent procedure

Participants will be recruited from the adult diabetes outpatient clinics at the University Hospital Bern or from referring clinicians working at other outpatient clinics. Potential participants will be identified by their treating clinicians. If agreed by the participants, their contact details will be shared with the research team who will approach them to provide further details about the study, both written and spoken. Specifically, the investigators will explain to each interested individual the nature of the study, its purpose, the procedures involved, the expected duration, the potential risks and benefits and any discomfort it may entail. Each participant will be informed that the participation in the study is voluntary and that he or she may withdraw from the study at any time and that withdrawal of consent will not affect his or her subsequent medical assistance and treatment. Participants will also be informed that there will be no reimbursement for the

participation in this investigation and that authorised individuals other than their treating physician may examine his/her medical records.
A study information sheet, which also contains the consent form, will be send out to the potential participants. Any questions from the subject or its related persons shall be addressed. Enough time will be given to the participant to decide whether to participate or not. Written consent will be obtained after the provision of a minimum of 24h of reflection time.
If continued interest exists and participants appear eligible, participants will be asked to sign the consent form and return it to the investigators before or on the day of the arranged baseline visit. The Sponsor-Investigator or its designate will sign the returned document before the commencement of the baseline visit and ask the participant whether a copy is desired. The participants will not be submitted to any study-related procedure before obtaining written informed consent. The signed consent form will be retained as part of the study records. The informed consent process will be documented in the patient file and any discrepancy to the process described in the protocol will be explained.
The number of approached patients who either refuse to participate in the trial or are not deemed suitable for participation by the clinical investigator will be documented with respective reason. Details will be used to create the CONSORT Flow Diagram at the end of the trial.

3.3 Study procedures

The study design is illustrated in Figure 1 and the schedule and details of study-related activities can be found in the Investigation Schedule (Appendix 1). To increase the convenience, all study visits can be held remotely using the Hospital's approved telemedicine channels or in-clinic visits when it coincides with the time of usual care appointments.

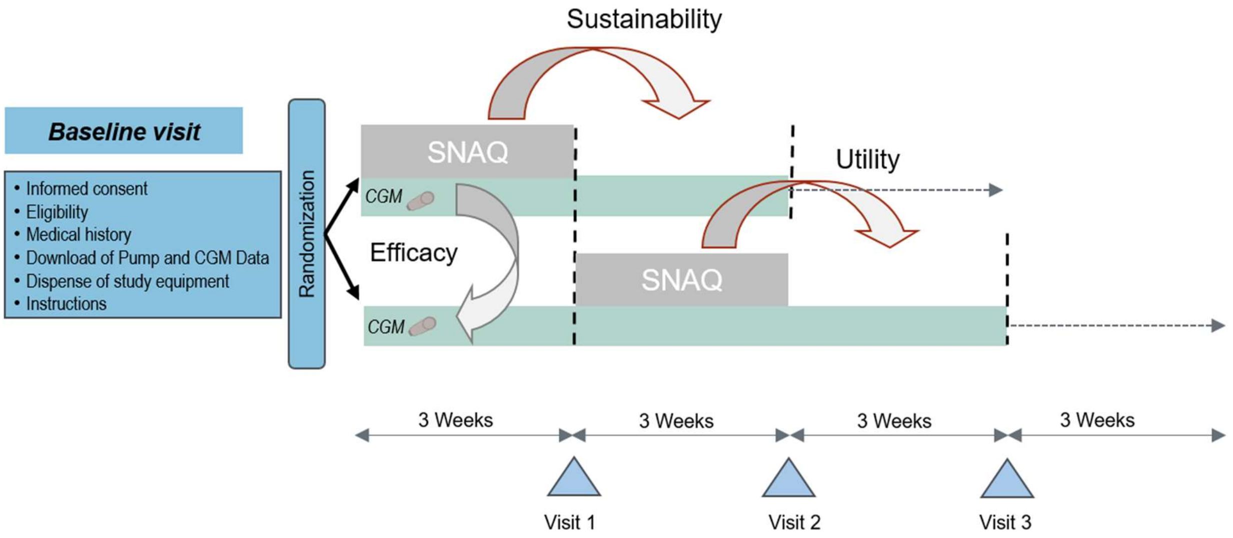

Figure 1. Study Flow

Baseline visit

The baseline visit will include the following procedure

- Retrieval of relevant medical information
- Questionnaire exploring the participants' current meal management practices, perceived importance and related burden
- Nutrition literacy questionnaire

In order to assess people's baseline glucose control and insulin requirements, participants will be asked to download their data from the hybrid closed-loop systems from the 14 preceding days and share them with the study team.

The following details will be recorded in the eCRF to characterize the population:

- Sociodemographic and anthropometrics variables (age, gender, body weight, height, level of education, socioeconomic status)
- Diabetes-specific medical details: age at diagnosis, duration of diabetes, type of commercial hybrid closed-loop system, type of insulin, diabetes-related complications
- Comorbidities
- Medications other than insulin (glucose lowering agents, lipid lowering agents, anti-hypertensive agents)
- History of severe hypoglycaemia and hypoglycaemia awareness status (Gold score)

All recorded parameters including units and scales are defined in the case report form.
In participants without a smartphone compatible with the newest version of SNAQ app, a study phone will be provided to the patients for the duration of the study. In addition, all participants will be provided with the premium version of SNAQ app for 12 months (including 6 weeks of the study and excluding the period when the usage of the app is not intended regarding the participation group) by using a specific code.
After completion of all baseline assessments participants will be randomized to the intervention or the control group. Participants of the intervention group will be asked to use SNAQ app for 3 weeks while in participants in the control group no instructions of any kind will be made by the study team. Before the use of the SNAQ app, participants of the intervention group will receive access to the premium version of the app using a specific code, assistance with the download of the app, registration with the assigned study ID and a short guidance through the app given by the team, using the informational material provided by SNAQ app.

Visit 1 (end of period 1)
Visit 1 will entail the following procedures:
- Acquisition of the data download from the closed-loop system
- Acquisition of the data download from the SNAQ app (intervention group only)
- Feedback questionnaire on the SNAQ app (intervention group only)
- Nutrition literacy questionnaire (intervention and control group)

At the end of the visit and throughout Period 2, participants in the intervention group will stop using SNAQ app. To ensure that participants of the intervention group will stop using the SNAQ app, the license of the app will be temporarily de-activated remotely. Before the use of the SNAQ app, participants of the control group will receive premium version of the app for 12 months (including 6 weeks of the study and excluding the period when the usage of the app is not intended regarding the participation group) assistance with the download of the app, registration with the assigned study ID and a short guidance through the app given by the team, using the informational material provided by SNAQ app.

Visit 2 (end of period 2)
Visit 2 will entail the following procedures:
- Acquisition of the data download from the closed-loop system
- Acquisition of the data download from the SNAQ app (control group only)
- Feedback questionnaire on the SNAQ app (control group only)

At the end of the visit and throughout period 3, participants of the control group will undergo a period of 3 weeks without using the SNAQ app. The license for using the SNAQ app will be temporarily de-activated remotely.

Visit 3 (control group only)
Visit 3 will entail the following procedures:
- Acquisition of the data downloads (data from the closed-loop system and the SNAQ app)

After the last visit (Visit 2 for participants of the intervention group and Visit 3 for the control group) participants will continue to have free access to the premium version of the SNAQ app. Participants will not receive instructions on how to use the app during this period. Data on SNAQ app use during this period will be collected at the end of the period. At the end of this period, the study will be terminated and the study phones (if applicable) will be collected.

During the whole study duration, all participants will continue to use their usual diabetes therapy (including hybrid-closed loop system and the type of insulin). The study will not specify nor change any aspects related to the participants' insulin treatment settings. Whenever possible, the participant should not change their diabetes therapy during the course of the study due to the potential of confounding effects.

Methods

Sensor glucose control, insulin dose and treatment settings

For each study period, sensor glucose, insulin data and treatment settings will be self-downloaded by the participants, de-identified (by replacing identifiable information by the assigned study ID) and shared with the study team. After checking that all data is de-identified, the study team will store the files as source data in the REDCap study base. Raw data will be further processed in R using custom scripts to calculate outcomes based on sensor glucose and insulin data. All signals will be resampled at 5 minutes intervals before further processing using linear interpolation. Sensor glucose gaps up to 20 minutes will be interpolated (gaps larger than 20 minutes will be discarded for the calculation of sensor glucose control). All calculated variables will be entered into the study database.

Following variables will be calculated or retrieved:

- Total daily insulin dose (daily basal and bolus dose, number of manual boluses)
- Carbohydrate (g/U), insulin sensitivity factors (mmol/L/U), active insulin time (h), glucose target (mmol/L)
- Sensor glucose control (time in target range, time in hypoglycaemia (time < 3.0mmol/L, time < 3.9 mmol/L, time between 3.1-3.9 mmol/L), time in hyperglycaemia (time > 10.0 mmol/L, time > 13.9 mmol/L, time between 10.1-13.9 mmol/L, time > 20.0 mmol/L), postprandial time in target range, postprandial time in hypoglycaemia (time < 3.0mmol/L and time < 3.9 mmol/L, time between 3.1-3.9 mmol/L), postprandial time in hyperglycaemia (time > 10.0 mmol/L, time > 13.9 mmol/L, time between 10.1-13.9 mmol/L, time > 20.0 mmol/L), mean sensor glucose, SD of sensor glucose, CV of sensor glucose)

Dietary intake

Daily frequency and mean daily amount and timing of macronutrient intake (carbohydrate, protein, fat) will be calculated from the download of the SNAQ app. Information on carbohydrate intake will be additionally calculated from data of the hybrid closed-loop system (carbohydrates entered by the participants in their closed-loop systems).

Nutrition literacy, meal management and feedback questionnaires

Nutrition literacy will be expressed as mean signed and absolute relative errors (%) of carbohydrate error and be calculated over all items of the quiz on a subject level. The nutrition literacy, the meal management and the feedback questionnaire will be implemented in the survey tool of the RedCap platform and can be completed remotely using a participant specific link.

Usability/Usefulness

The frequency of SNAQ app use will be calculated for the applicable study periods. Further objective information on the usability of the SNAQ app will be evaluated by assessing the manual corrections of the segmentation, food recognition and proposed meal macronutrient content. All data will be retrieved from the SNAQ app. The System Usability Scale will be filled by the participants after the intervention to assess the usability of the app as part of the feedback questionnaire.

3.4 Withdrawal and discontinuation

Subjects will be withdrawn from the investigation under the following circumstances

- The subject's behavior interferes with a safe conduct of the study
- Decision by the treating clinical team that termination is in the subject's best medical interest
- Decision by the Sponsor-investigator that termination is in the subjects' best medical interest
- The subject wishes to terminate the study
- Pregnancy during the study period, as confirmed by a positive urine-pregnancy test (beta-hCG elevation over test-specific threshold).

Participants who are withdrawn from the study will not be replaced. Reasons for withdrawal (if indicated) will be documented. In case of withdrawal upon subject request, all collected data remains coded, as indicated in Informed Consent document. Efforts will be made to follow the subject up for the primary endpoint assessment even if the intervention is discontinued. Due to the nature of the study no exit survey/examination will be performed in participants who discontinue the participation in the study prior to the final visit unless for safety reasons.

3.5 Randomization

Eligible participants will be randomly allocated (1:1) to the intervention group or the control group. Group allocation will be done according to an adaptive stratified randomization framework using the minimization method, implemented in the MinimPy randomisation software to balance between group characteristics. The following minimisation factors will be considered: glycated haemoglobin A1C (<7.5 or ≥7.5 %), sex, time in target range (3.9-10.0mmol/L) in the 14 days prior to the study inclusion (>75% or <75%).

4 STATISTICS AND METHODOLOGY

4.1. Statistical analysis plan and sample size calculation

Sample Size calculation

Sample size was estimated to provide sufficient power to detect a clinically meaningful difference of 10% in the primary efficacy outcome. The sample size was calculated based on preliminary data on the efficacy of SNAQ app in improving postprandial glucose control. In data from 23 subjects (1603 meals with SNAQ app and 2018 meals without SNAQ app), mean±SD time in target range was 85±23% vs. 77±35% with and without the app, respectively. Assuming a mean improvement of 10% in the intervention group with no change in the control group, and assuming

a within-participant standard deviation of 10% in both groups, a total sample size of 34 participants (17 per group) is required to achieve a power of 80% at a two-sided alpha level of 5%. Assuming a drop-out rate of 20%, we therefore aim to recruit 44 participants (22 per group).

Statistical analysis

For the efficacy analysis, outcomes will be compared between the two groups using general linear models (GLM). All endpoints evaluating the efficacy of the intervention (main analysis) will be adjusted for their values at baseline. For the sustainability analysis, baseline data and the 3 weeks following SNAQ app discontinuation will be compared using pooled data from both groups. GLM techniques considering the paired data structure will be used. In case of non-normality of the residuals, data will be transformed appropriately. All analyses will be performed using all available data (see section 4.2 for handling of missing data). P-values below 5% will be considered statistically significant. Exploratory endpoints will be reported as point estimates with 95% confidence intervals (unadjusted for multiple testing), so definite inferences cannot be made regarding those endpoints.
Statistical analysis will be performed using the software R and/or Stata. A detailed statistical analysis plan will be written before the start of the study.

4.2. Handling of missing data and drop-outs

The number of missing values for all outcomes for each treatment condition will be reported. There will be no imputation for missing data. For each patient, outcomes calculated from continuously recorded data (e.g. time in sensor glucose ranges) will be calculated if at least 30% of the data of the respective period are available. If less than 30% of data is available, the respective data will be discarded.

5 REGULATORY ASPECTS AND SAFETY

5.1 Local regulations / Declaration of Helsinki

This study is conducted in compliance with the protocol, the current version of the Declaration of Helsinki, the ICH-GCP, the HRA as well as other locally relevant legal and regulatory requirements(1).

5.2 (Serious) Adverse Events and notification of safety and protective measures

An Adverse Event (AE) is any untoward medical occurrence in a patient or a clinical investigation subject which does not necessarily have a causal relationship with the trial procedure. An AE can therefore be any unfavorable or unintended finding, symptom, or disease temporally associated with a trial procedure, whether or not related to it.

A Serious Adverse Event (SAE) (ClinO, Art. 63) is any untoward medical occurrence that

- Results in death or is life-threatening,
- Requires in-patient hospitalization or prolongation of existing hospitalization,
- Results in persistent or significant disability or incapacity, or
- Causes a congenital anomaly or birth defect

Both Investigator and Sponsor-Investigator make a causality assessment of the event to the trial intervention, (see table below based on the terms given in ICH E2A guidelines). Any event assessed as possibly, probably or definitely related is classified as related to the trial intervention.

Relationship	Description
Definitely	Temporal relationship<br>Improvement after dechallenge*<br>Recurrence after rechallenge<br>(or other proof of drug cause)
Probably	Temporal relationship<br>Improvement after dechallenge<br>No other cause evident
Possibly	Temporal relationship<br>Other cause possible
Unlikely	Any assessable reaction that does not fulfil the above conditions
Not related	Causal relationship can be ruled out
*Improvement after dechallenge only taken into consideration, if applicable to reaction	

Both Investigator and Sponsor-Investigator make a severity assessment of the event as mild, moderate or severe. Mild means the complication is tolerable, moderate means it interferes with daily activities and severe means it renders daily activities impossible.

Device deficiencies (DD) and all **adverse events (AE)** including all **serious adverse events (SAE)** are collected, fully investigated and documented in the eCRF during the entire investigation period, i.e. from patient's informed consent until the last study-specific procedure, including a safety follow-up period (if applicable). Documentation of AEs (including SAEs) by the Sponsor-Investigator, Principal Investigator (or authorized designee) includes diagnosis or symptoms, start and stop dates of event, event treatment, event resolution, assessment of seriousness and causal relationship to the trial intervention.

Reporting of SAEs (see ClinO, Art. 63)

All SAEs are documented and reported immediately (within a maximum of 24 hours) to the Sponsor-Investigator of the study.

If it cannot be excluded that the SAE occurring in Switzerland is attributable to the intervention under investigation, the Investigator reports it to the Ethics Committee via BASEC within 15 days.

Follow up of (Serious) Adverse Events

The information on (Serious) AEs is systematically collected at the regular visits, as applicable and clinically justified in the context of the specific study setting. Ongoing (serious) Adverse Events will be monitored and documented until event resolution. A standardized follow-up period after study completion or withdrawal, in the absence of study-related safety events, is not deemed necessary, as any untoward, delayed and health-relevant study-related incident are considered unlikely.

Notification of safety and protective measures (see ClinO, Art 62, b)

If immediate safety and protective measures have to be taken during the conduct of the study, the investigator notifies the Ethics committee of these measures, and of the circumstances necessitating them, within 7 days.

5.3 (Periodic) safety reporting

An annual safety report (ASR) will be submitted once a year to the local Ethics Committee by the Sponsor-Investigator (ClinO, Art. 43 Abs 1).

5.4 Pregnancy

Eligibility assessment will not include a pregnancy test as the study intervention (use of a food analysis app) is not contra-indicated during pregnancy and might be even endorsed. However, pregnant women shall be excluded due to concerns regarding data interpretability. To avoid unnecessary burden on the participants, self-reported negative pregnancy status will be considered sufficient. In addition, participants will be encourage to use a contraceptive method during the time of the study and notify the study team if pregnancy still occurs. Pregnant participant will be withdrawn from the study due to concerns related to data validity.

5.5 Amendments

Substantial changes to the study setup and study organization, the protocol and relevant study documents are submitted to the Ethics Committee for approval before implementation. Under emergency circumstances, deviations from the protocol to protect the rights, safety and well-being of human subjects may proceed without prior approval of the Ethics Committee. Such deviations shall be documented and reported to the Ethics Committee as soon as possible.

Substantial amendments are changes that affect the safety, health, rights and obligations of participants, changes in the protocol that affect study objective(s) or central research topic, changes of study site(s) or of study leader and sponsor (ClinO, Art. 29).

A list of all non-substantial amendments will be submitted once a year to the Ethics Committee together with the ASR.

5.6 Notification and reporting upon completion, discontinuation or interruption of the study

Upon regular study completion, the Ethics Committee is notified via BASEC within 90 days (ClinO, Art. 38).

The Sponsor-Investigator may terminate the study prematurely according to certain circumstances, e.g.

- Ethical concerns,
- Insufficient participant recruitment,
- When the safety of the participants is doubtful or at risk (e.g. when the benefit-risk assessment is no longer positive),
- Alterations in accepted clinical practice that make the continuation of the study unwise, or
- Early evidence of harm or benefit of the experimental intervention

Upon premature study termination or study interruption, the Ethics Committee is notified via BASEC within 15 days (ClinO, Art. 38).

All health-related data will be stored for a total of 10 years after study termination or premature termination of the study.

A final report is submitted to the Ethics Committee via BASEC within a year after completion or discontinuation of the study, unless a longer period is specified in the protocol (ClinO, Art. 38).

5.7 Insurance

The present study represents a risk Category A1 investigation and is therefore exempt from study insurance policies (Art. 3 ClinO-MD).

6 FURTHER ASPECTS

6.1 Overall ethical considerations

Approval of the lead Ethics Committee will be obtained before the commencement of any study-related activities. Additional requirements set by the authorities will be implemented throughout the study.

The study procedures do not expose participants to any additional risk or invasive procedures. Apart from using the SNAQ app and undergoing the planned remote or in-person visits, participation in the trial does not impose additional burden on the patients. Meal management apps are widely used in the population. The SNAQ app has been on the market for several years and currently counts more than 40,000 users (with over 2,500 users in Switzerland).

Although real life data from an observation study involving people with type 1 diabetes were presented at the Diabetes Technology Meeting 2021, we are not aware of any randomized clinical trial assessing the benefits of SNAQ app on various aspects of type 1 diabetes people's live (e.g. glucose control, satisfaction with the app, food literacy, etc.). Given the wide-spread use of SNAQ app in the diabetes community and several endorsements of the app on digital media, we feel that an objective assessment through the use of a proper study design and statistical techniques, conducted by a purely academic research team without any conflicts of interest, could provide people with type 1 diabetes with a better basis for decision-making of meal management tools such as SNAQ app. This necessity was also perceived by patient public involvement contributors.

6.2 Risk-benefit assessment

One of the most challenging aspects of managing type 1 diabetes is meal planning and assessment, in particular carbohydrate counting, which takes up a lot of time and efforts in people's daily life. Although nutritional values (e.g. carbohydrate content per standard serving) can be accessed by various tools (e.g. libraries, exchange tables), estimation of portion sizes often relies on the people's visual estimation as weighing food is often not a viable or convenient option in daily life. Outcomes of patient survey suggest that carbohydrate counting significantly contributes to the burden of type 1 diabetes self-management and that there is a general hope that new technology could lower it. By using SNAQ app, participants are provided with a simple method that may aid them to estimate carbohydrate contents with greater accuracy and hence improve postprandial control. Apart from the potentially positive influence of the food analysis app on glucose control and burden of self-management, the app has the potential to enhance participants' capacity to access nutritional information, increase health- and nutrition-related knowledge as well skills in preparing well-balanced meals of high nutritional quality. Increased food literacy and self-efficacy is an established predictor for the prevention of cardiovascular complications.

In terms of risk, previous work suggest that the SNAQ app estimates food carbohydrate content with greater accuracy than the average patient [9]. In addition, the food analysis app, SNAQ, only supports the estimation of carbohydrate content, but the final decision in terms of treatment decisions lies with the participants. As a consequence, the use of the app is by no means related to higher risk than when counting carbohydrate by experience or other methods of choice. We therefore interpret the risk-benefit-ratio in favour of the benefits.

7 QUALITY CONTROL AND DATA PROTECTION

7.1 Quality measures

For quality assurance the sponsor, the Ethics Committee or an independent trial monitor may visit the research sites. Direct access to the source data and all study related files is granted on such occasions. All involved parties keep the participant data strictly confidential. Study personnel will be trained on all important study related aspects. Quality measures will follow the standardized

procedures of the University Clinic for Diabetes, Endocrinology, Nutritional Medicine and Metabolism which include an internal review of the procedures and a check to the adherence to good clinical practice guidelines. An independent data review by a trained data manager will be performed and all data will be checked for plausibility.

7.2 Data recording and source data

All study data will be collected in an electronic Case Report Form (eCRFs) or downloaded directly from the devices. Data from the participants' usual care hybrid closed-loop systems will be de-identified and coded with the Study ID. The imported data from the SNAQ app is already de-identified because they will be logged-in with their study ID. All data will be archived in a coded format in the study database RedCap with the exception of signed informed consent forms which will be stored in a locked cabinet.

During the study, eCRFs will be maintained and kept current by the study team. eCRFs are linked with participants' study ID. The study delegation log describes who will be authorized for eCRF entries and access to the data. Once data collection is completed and validated, the Sponsor-Investigator will sign off all eCRFs. The Sponsor-investigator is responsible for the correctness of the entered data and confirms this by means of the electronic signature in the RedCap database. The data management plan specifies what constitutes source data. In case eCRFs are not serving as source documents, source documents will be retained for audit trail purposes. Location of source data is specified in the data management plan.

Source documents in this trial entail the following:

- Signed and dated informed consent forms
- Randomisation logs
- Insulin and CGM data exported from the closed-loop systems (via the proprietary platforms such as Glooko or Carelink)
- Meal macronutrient contents and timing exported from the SNAQ app.
- eCRFs on which data are entered directly

Any change or correction to source data will be dated, marked with staff initials, and explained (if necessary) and should not obscure the original entry.

All data captured in the eCRF will be itemised on a source data location list, which will be stored in the investigator site file. This list should clearly indicate the source data location corresponding to each eCRF entry. If several sources are possible for one eCRF entry, the priority order of these will be specified in the list. If certain data are directly entered into the eCRF (and are thus considered as source data) this will be specified on the location list accordingly.

Note: If the participants send the data without de-identifying them beforehand, the study team will de-identify data before uploading the data in REDCap.

7.3 Confidentiality and coding

Trial and participant data will be handled with uttermost discretion and is only accessible to authorised personnel who require the data to fulfil their duties within the scope of the study. On the CRFs and other study specific documents, participants are only identified by a unique study ID.

All collected data will be labelled with the subject ID (composed of the study acronym and a two digit number; e.g. SuMMIT_01). No biological specimens will be collected. The subject identification list will be kept in the Investigator Site File during the course of the clinical trial. During the study the Sponsor-Investigator has access to all documents of the investigation including randomization lists and coding keys.

After completion or termination of the study, the subject identification list will be kept by a person outside of the study team (Roman Trepp, Department of Diabetes, Endocrinology Nutritional Medicine and Metabolism, University Hospital Bern). In case of further use of research data, the researchers in charge will not have access to the subject identification list.
Direct access to source documents will be permitted for purposes of monitoring, audits and inspections. The Sponsor-Investigator will declare who will have access to the documents of the investigation, dataset, randomization code, etc. during and after the investigation.

The study data base in REDCap® can only be accessed by designated investigator staff entering a username and password. The application has a group and role-based security model. Each user belongs to one or more security groups with specific sets of permissions about folder or projects in the system. Only dedicated site administrators have access to the admin console, enabling user management and changing security settings.

All events are recorded in the user event list of the audit log files. Data are stored and visualised in data grids either in the format of datasets, lists or assays. Each change of data is tracked and documented in corresponding audit log files.
The servers are behind a firewall and cannot be accessed through the internet. They are located in locked dedicated server rooms with restricted access. Apache HTTP Server and REDCap® were configured to run under Secure Sockets Layer (SSL) which implies that data is encrypted and transmitted securely.
Available disk space is monitored actively. If free disk space is less than 10%, administrators get an email, and more storage capacity will be added accordingly.
All servers are regularly backed up on storage servers in a separate server room using a multi-level system.

7.4 Retention of study data

Paper-based trial documents (e.g. Informed Consent) will be adequately stored within the facilities of the Department of Diabetes, Endocrinology, Nutritional Medicine and Metabolism for at least 10 years after completion of the study. The study database will be securely stored at Department of Diabetes, Endocrinology, Nutritional Medicine and Metabolism, Inselspital, Bern and the retention duration of study documents will follow local legal requirements.
If subjects provided consent for the further use of their data (independently of the investigation specific consent), data will be stored in the same database for an undetermined length of time and will be accessible for reuse in the context of other research projects. No biological samples will be collected.

8 MONITORING AND REGISTRATION

The Sponsor-Investigator will ensure oversight of the clinical research study by means of pre-defined monitoring visits. The level and type of monitoring will be commensurate with the Swiss Clinical Trial organization SCTO Risk Monitoring Score of 2 corresponding to a low risk. Source data and all project related files and documents will be made accessible to monitors. The Sponsor-Investigator and site staff will be available and ready to answer questions that may arise during the monitoring. Details will be specified in a dedicated monitoring plan.
The study will be registered in the Swiss National Clinical trial Portal (SNCTP via BASEC). In addition, the study will be registered in clinicaltrials.gov, a registry listed in the WHO International Clinical Trials Registry Platform.

9. FUNDING / PUBLICATION / DECLARATION OF INTEREST

The project was granted seed funding from the European Foundation for the Study of Diabetes (EFSD, call on Digital Diabetes).

Study findings will be disseminated to study participants, healthcare professionals, the public, and scientist via summaries in lay language, scientific presentations and peer-reviewed publications. The privacy of each subject and confidentiality of their information shall be preserved in reports and publication of data. Disseminations will be detailed in a publication plan. If sex or gender effects are observed, they will be published in the final study report. Once results have been published, trial data will be accessible to external researchers. Investigators wishing to replicate the analyses or to do an individual patient meta-analysis may request the data from the Sponsor-Investigator. The investigators do not declare any conflict of interest.

10. REFERENCES

1. Rabasa-Lhoret, R., et al., *Effects of meal carbohydrate content on insulin requirements in type 1 diabetic patients treated intensively with the basal-bolus (ultralente-regular) insulin regimen.* Diabetes Care, 1999. **22**(5): p. 667-73.
2. Brazeau, A.S., et al., *Carbohydrate counting accuracy and blood glucose variability in adults with type 1 diabetes.* Diabetes Res Clin Pract, 2013. **99**(1): p. 19-23.
3. Alfonsi, J.E., et al., *Carbohydrate Counting App Using Image Recognition for Youth With Type 1 Diabetes: Pilot Randomized Control Trial.* JMIR Mhealth Uhealth, 2020. **8**(10): p. e22074.
4. Fortin, A., et al., *Practices, perceptions and expectations for carbohydrate counting in patients with type 1 diabetes - Results from an online survey.* Diabetes Res Clin Pract, 2017. **126**: p. 214-221.
5. Mozzillo, E., et al., *Unhealthy lifestyle habits and diabetes-specific health-related quality of life in youths with type 1 diabetes.* Acta Diabetol, 2017. **54**(12): p. 1073-1080.
6. Lawton, J., et al., *Dose Adjustment for Normal Eating: a qualitative longitudinal exploration of the food and eating practices of type 1 diabetes patients converted to flexible intensive insulin therapy in the UK.* Diabetes Res Clin Pract, 2011. **91**(1): p. 87-93.
7. Corbin, K.D., et al., *Obesity in Type 1 Diabetes: Pathophysiology, Clinical Impact, and Mechanisms.* Endocr Rev, 2018. **39**(5): p. 629-663.
8. Lawton, J., et al., *The impact of using a closed-loop system on food choices and eating practices among people with Type 1 diabetes: a qualitative study involving adults, teenagers and parents.* Diabet Med, 2019. **36**(6): p. 753-760.
9. Herzig, D., et al., *Volumetric Food Quantification Using Computer Vision on a Depth-Sensing Smartphone: Preclinical Study.* JMIR Mhealth Uhealth, 2020. **8**(3): p. e15294.

Appendix 1: Schedule of assessments and procedures

Investigation period	Screening	Baseline visit (V0)	Visit 1	Visit 2	Visit 3	End of study
			---------------------------------- *12 weeks*	--------------------------------		
**Information about the study**	*G1,G2*	G1,G2				
**Written informed consent**		G1, G2				
**Eligibility screening**	G1, G2	G1, G2				
**Details of medical history and current diabetes treatment**		*G1, G2*	*G1, G2 (current diabetes treatment only)*	*G1, G2 (current diabetes treatment only)*	*G1, G2 (current diabetes treatment only)*	
**Anthropometry & sociodemographics**		*G1, G2*				
**Randomisation**		*G1, G2*				
**SNAQ instruction**		*G1*	*G2*			
**Meal management questionnaire**		*G1, G2*				
**Nutrition literacy questionnaire**		*G1, G2*	*G1, G2*			
**Feedback questionnaire**			*G1*	*G2*		
**Data download (SNAQ, hybrid closed-loop pump)**		*G1, G2*	*G1, G2*	*G1, G2*	*G1, G2*	*G2 (SNAQ data only)*
**Return of smartphone (if applicable)**					+	+
**Safety assessment**			+	+	+	+

G1: Intervention Group (SNAQ in Period 1); G2: Control Group
